# Single-year change in views of democracy and society and support for political violence in the USA: findings from a 2023 nationally representative survey

**DOI:** 10.1186/s40621-024-00503-7

**Published:** 2024-05-21

**Authors:** Garen J. Wintemute, Sonia L. Robinson, Andrew Crawford, Elizabeth A. Tomsich, Paul M. Reeping, Aaron B. Shev, Bradley Velasquez, Daniel Tancredi

**Affiliations:** 1 UC Davis Violence Prevention Research Program, Sacramento, CA USA; 2 Department of Emergency Medicine, School of Medicine, UC Davis, Sacramento, CA USA; 3 California Firearm Violence Research Center, Sacramento, CA USA; 4 Department of Pediatrics, UC Davis, Sacramento, CA USA

**Keywords:** Political violence, Firearm violence, Violence and society, Racism, Domestic violent extremism, Civil war, QAnon

## Abstract

**Background:**

A 2022 survey in the USA found concerningly high prevalences of support for and personal willingness to engage in political violence, of beliefs associated with such violence, and of belief that civil war was likely in the near future. It is important to determine the durability of those findings.

**Methods:**

Wave 2 of a nationally representative cohort survey was conducted May 18-June 8, 2023; the sample comprised all respondents to 2022’s Wave 1. Outcomes are expressed as weighted proportions; changes from 2022 to 2023 are for respondents who participated in both surveys, based on aggregated individual change scores.

**Results:**

The completion rate was 84.2%; there were 9385 respondents. After weighting, 50.7% (95% confidence interval (CI) 49.4%, 52.1%) were female; weighted mean (SD) age was 48.5 (25.9) years. About 1 in 20 respondents (5.7%, 95% CI 5.1%, 6.4%) agreed strongly/very strongly that “in the next few years, there will be civil war in the United States,” a 7.7% decrease.

In 2023, fewer respondents considered violence to be usually/always justified to advance at least 1 of 17 specific political objectives [25.3% (95% CI 24.7%, 26.5%), a 6.8% decrease]. However, more respondents thought it very/extremely likely that within the next few years, in a situation where they consider political violence justified, “I will be armed with a gun” [9.0% (95% CI 8.3%, 9.8%), a 2.2% increase] and “I will shoot someone with a gun” [1.8% (95% CI 1.4%, 2.2%), a 0.6% increase]. Among respondents who considered violence usually/always justified to advance at least 1 political objective, about 1 in 20 also thought it very/extremely likely that they would threaten someone with a gun (5.4%, 95% CI 4.0%, 7.0%) or shoot someone (5.7%, 95% CI 4.3%, 7.1%) to advance such an objective.

**Conclusions:**

In this cohort, support for political violence declined from 2022 to 2023, but predictions of firearm use in political violence increased. These findings can help guide prevention efforts, which are urgently needed.

**Supplementary Information:**

The online version contains supplementary material available at 10.1186/s40621-024-00503-7.

## Background

In the USA, experts both inside and outside of government have documented an increase in incidents of political violence (the use of force or violence to advance political objectives) (Armed Conflict Location Event Data Project 2019) and have issued repeated warnings about the potential for such violence to disrupt democratic processes in America and jeopardize Americans’ health and safety (Kleinfeld 2021; Walter 2022; Kalmoe and Mason 2022; Armed Conflict Location Event Data Project 2022; Federal Bureau of Investigation and Department of Homeland Security 2022; Office of the Director of National Intelligence 2021; House Select Committee to Investigate the January 6th Attack on the United States Capitol 2022). Political violence is a public health problem.

In 2022, we conducted Wave 1 of a nationally representative cohort survey exploring Americans’ support for and willingness to engage in political violence and the prevalence of beliefs associated with such violence. The first report (Wintemute et al. 2024) from that survey presented concerning findings for the population as a whole, among them that nearly one-third of Americans (32.8%) considered violence usually or always justified to advance at least 1 of 17 specified political objectives and that 13.7% strongly or very strongly agreed with the statement that “in the next few years, there will be civil war in the United States.” Additional reports have examined variation in these and other measures with party affiliation and political ideology (Wintemute et al. 2022) and focused on particular populations of interest: MAGA Republicans (Wintemute et al. 2024); firearm owners (Wintemute et al. 2024); and those who hold racist beliefs, endorse the use of violence to effect social change, or approve of specific extremist organizations and movements that have been linked to violence (Wintemute et al. 2023).

This study presents initial findings from Wave 2 of the survey, conducted in mid-2023 among respondents to Wave 1. The focus of the analysis is on change from 2022 to 2023 in the findings presented in our first report (Wintemute et al. 2023), based on linked observations for 9385 participants who responded in both years. We also expand our findings on predicted firearm use in political violence, assessing differences between respondents who do and do not consider political violence justifiable.

## Methods

Methods for Wave 2 of this cohort survey closely followed those for Wave 1 (Wintemute et al. 2023). Wave 2 was designed by the authors and administered online in English and Spanish from May 18 to June 8, 2023 by the survey research firm Ipsos (Ipsos 2021a). The study was reviewed by the University of California Davis Institutional Review Board (protocol 187125: exempt from full review, category 2, survey research). Before participants accessed the questionnaire, they were provided informed consent language that concluded, “[by] continuing, you are agreeing to participate in this study.” The study is reported following American Association for Public Opinion Research guidelines (American Association for Public Opinion Research 2021).

### Participants

Participants for Wave 1 were drawn from the Ipsos KnowledgePanel, an online research panel that has been widely used in population-based research on violence and firearm ownership (Kravitz-Wirtz et al. 2021; Wintemute et al. 2022; Schleimer et al. 2020; Miller et al. 2022; Miller and Azrael 2022; Salhi et al. 2020). To establish a nationally representative panel, KnowledgePanel members are recruited on an ongoing basis through address-based probability sampling using data from the US Postal Service’s Delivery Sequence File (Ipsos. Knowledge 2021b). Recruitment into KnowledgePanel involves repeated contact attempts, if necessary, by mail and telephone. Recruited adults in households without internet access are provided a web-enabled device and free internet service, and a modest, primarily points-based incentive program seeks to encourage participation and promote participants’ retention in KnowledgePanel over time (Ipsos. Knowledge. 2021b).

A probability-proportional-to-size procedure was used to select a study-specific sample for Wave 1. All panel members who were aged 18 years and older were eligible for selection. Invitations were sent by e-mail; automatic reminders were delivered to non-respondents by e-mail and telephone beginning 3 days later (Ipsos 2021a).

The Wave 1 survey was conducted May 13 to June 2, 2022. It included a main sample, which had a completion rate of 53% and provided the study population for our initial report (Wintemute et al. 2023), and oversamples of firearm owners, transgender people, combat veterans, and California residents that were recruited to ensure adequate power for planned analyses. Compared with main sample nonrespondents, main sample respondents were older and more frequently white, non-Hispanic; were more often married; had higher education and income; and were less likely to be working (Wintemute et al. 2023).

Including the main sample and oversamples, Wave 1 comprised 12,947 respondents. Of those respondents, 11,140 (86.0%) remained active members of KnowledgePanel on Wave 2’s launch date and were invited to participate in Wave 2. (The 1807 Wave 1 respondents who were not active members of KnowledgePanel on Wave 2’s launch date had left the cohort through normal attrition.)

A final Wave 2 survey weight variable provided by Ipsos adjusted for the initial probability of selection into KnowledgePanel and for survey-specific nonresponse and over- or under-coverage using design weights with post-stratification raking ratio adjustments. As with the 2022 sample, the weighted 2023 sample is designed to be statistically representative of the noninstitutionalized adult population of the USA as reflected in the 2021 March supplement of the Current Population Survey (Ipsos. 2021a).

### Measures

Sociodemographic data were collected by Ipsos from profiles created and maintained by KnowledgePanel members. Survey questions that supplied data for this analysis covered 3 broad domains: beliefs regarding democracy and the potential for violence and civil war in the USA, beliefs regarding American society and institutions, and support for and willingness to engage in political violence.

Our primary outcome measures again concerned political violence. Violence was represented by the phrase “force or violence,” defined in the questionnaire as “physical force strong enough that it could cause pain or injury to a person.” “Force or violence to advance an important political objective that you support” was used in questions about respondents’ support for and willingness to engage in political violence.

As in 2022, respondents were asked about the extent to which they considered political violence to be justified “in general” and then about justification for its use to advance specified political objectives. Example objectives include “to return Donald Trump to the presidency this year,” “to preserve an American way of life based on Western European traditions,” and “to stop police violence” (Tables 6 and 7). Responses for 17 objectives were collected in both years. In 2022, 9 of 17 were presented to all respondents and 8 were paired, with respondents randomized for each pair to see 1 item; each respondent was presented with 13 of 17 objectives. In 2023, all 17 items were presented to all respondents.

Respondents who considered political violence to be at least sometimes justified for at least 1 of these 17 objectives in 2023 were asked about their personal willingness to engage in political violence: by type of violence (to “damage property,” “threaten or intimidate a person,” “injure a person,” “kill a person”) and by target population (examples: “an elected federal or state government official,” “a police officer,” “a person who does not share your religion”) (Tables 8 and 9).

All respondents were asked about the likelihood of their future use of firearms in a situation where they consider political violence to be justified (examples: “I will be armed with a gun,” “I will shoot someone with a gun”) (Table 10).

The full text of all questions reported on here, including sources for questions from prior surveys by other investigators, is in the Supplement (see Additional file 1).

### Implementation

Ipsos translated the questionnaire into Spanish, and interpreting services staff at UC Davis Medical Center reviewed the translation. Thirty-three KnowledgePanel members participated in a pretest of the English language version that was administered May 5–9, 2023.

Respondents were randomized 1:1 to receive response options in order from either negative to positive valence (example: from ‘do not agree’ to ‘strongly agree’) or the reverse throughout the questionnaire. Where a question presented multiple statements for respondents to consider, the order in which those statements were presented was randomized unless ordering was necessary. Logic-driving questions (those to which responses might invoke a skip pattern) included non-response prompts.

We employed unipolar response arrays without a neutral midpoint (e.g., do not agree, somewhat agree, strongly agree, very strongly agree). The literature is not in agreement on whether such midpoints should be included (Chyung et al. 2017; Westwood et al. 2022). We were persuaded by the studies reviewed by Chyung et al. (Chyung et al. 2017), which suggest that such midpoints allow respondents to choose “a minimally acceptable response as soon as it is found, instead of putting effort to find an optimal response,” a behavior known as satisficing. According to those authors, satisficing is particularly common when respondents are uncomfortable with the topics of the survey or under social desirability pressures, and both conditions apply here. Our analyses focus on responses above the “somewhat” or “sometimes” level to minimize the impact of potential satisficing on the results.

### Statistical Analysis

To generate prevalence estimates, we calculated weighted percentages and 95% confidence intervals (CI) using PROC SURVEYMEANS in SAS version 9.4 (SAS Institute, Inc., Cary, NC) and Complex Samples Frequencies in IBM SPSS Statistics, version 29 (IBM Corp., Armonk, NY).

Each survey item was ordinal and was subject to non-response. We report weighted frequencies for each item for each possible response. In addition, we summarized each item’s non-missing responses for a given year by assigning integer values to ordinal levels to produce an item score and then averaging them.

To rigorously describe between-year changes in survey responses, we accounted for the longitudinal study design by computing within-individual change scores and then summarizing those. To compute differences in percentage choosing a particular response, we created indicator variables for each year for each item and each possible response and then computed the within-individual change score between the two survey years for each item and response level. To compute differences in mean response scores, we computed within-individual change scores for the item scores, restricted to the sample of respondents with non-missing responses to the item in both years. Between-year comparisons on whether respondents considered violence justified for at least 1 of the 17 specified political objectives were restricted for each respondent to the 13 items presented to that respondent in 2022.

Tables present findings for all respondents (main sample and oversamples) in 2022 and 2023, mean differences from 2022 to 2023 for each response option, mean item scores, and mean differences in item scores.

## Results

Of 11,140 panel members invited to participate as part of the main study sample, 9385 completed the survey, yielding an 84.2% completion rate. The median survey completion time was 25 min (interquartile range, 18.6 min). Item non-response for items included in this analysis ranged from 0.5% to 4.0%; only 3 items had non-response percentages above 3.0% (see Supplement, Additional file 1).

After weighting, half of the respondents (50.7%, 95% CI 49.4%, 52.1%) were female; 62.7% (95% CI 61.2%, 64.1%) were white, non-Hispanic (Table 1). The weighted mean (SD) respondent age was 48.5 (25.9) years. Nonrespondents were younger than respondents (mean (SD) ages 52.5 (17.5) and 57.0 (16.5)) and less frequently male and white, non-Hispanic (Table S1).

**Table 1:** Personal characteristics of respondents

Characteristic	2022 Respondents (*n* = 12,947)		2023 Respondents* (*n* = 9385)	
	Unweighted *n*	Weighted % (95% CI)	Unweighted *n*	Weighted % (95% CI)
Age				
18–24	488	10.5 (9.6, 11.4)	310	10.3 (9.2, 11.5)
25–34	1309	16.4 (15.5, 17.4)	856	16.8 (15.6, 18.0)
35–44	1884	18.5 (17.7, 19.4)	1252	18.5 (17.4, 19.6)
45–54	1847	14.5 (13.8, 15.2)	1255	14.3 (13.4, 15.2)
55–64	2794	17.5 (16.8, 18.2)	2043	17.6 (16.7, 18.5)
65–74	2952	14.4 (13.8, 15.1)	2342	14.5 (13.8, 15.3)
75 +	1673	8.1 (7.6, 8.6)	1327	8.0 (7.4, 8.5)
Non-response	0	0.0 (0.0, 0.0)	0	0.0 (0.0, 0.0)
Gender				
Female	5652	50.7 (49.6, 51.8)	3866	50.7 (49.4, 52.1)
Male	7028	47.2 (46.1, 48.3)	5340	47.0 (45.7, 48.4)
Transgender	74	0.5 (0.4, 0.7)	45	0.5 (0.3, 0.7)
Non-binary	91	0.7 (0.5, 0.9)	59	0.8 (0.5, 1.0)
Other	24	0.2 (0.1, 0.3)	21	0.3 (0.1, 0.5)
Non-response	78	0.7 (0.5, 0.9)	54	0.7 (0.4, 0.9)
Race/Ethnicity				
White, Non-Hispanic	9491	62.6 (61.5, 63.8)	7014	62.7 (61.2, 64.1)
Black, Non-Hispanic	1095	11.9 (11.1, 12.7)	748	12.0 (10.9, 13.0)
Hispanic, any race	1504	16.9 (15.9, 17.8)	1016	16.9 (15.7, 18.1)
American Indian or Alaska Native, Non-Hispanic	76	1.2 (0.8, 1.5)	47	1.1 (0.7, 1.5)
Asian American or Pacific Islander, non-Hispanic	393	5.5 (4.8, 6.1)	277	5.5 (4.7, 6.2)
Some other race, Non-Hispanic	25	0.1 (0.1, 0.2)	19	0.1 (0.1, 0.2)
2 + Races, Non-Hispanic	363	1.8 (1.5, 2.0)	264	1.8 (1.4, 2.2)
Non-response	0	0.0 (0.0, 0.0)	0	0.0 (0.0, 0.0)
Marital status				
Now married	8074	56.1 (55.0, 57.3)	5961	56.2 (54.8, 57.6)
Widowed	770	4.1 (3.7, 4.5)	582	3.9 (3.5, 4.4)
Divorced	1456	8.7 (8.2, 9.2)	1010	8.2 (7.6, 8.8)
Separated	193	1.7 (1.4, 2.0)	122	1.4 (1.1, 1.8)
Never married	2454	29.4 (28.2, 30.5)	1710	30.2 (28.8, 31.6)
Non-response	0	0.0 (0.0, 0.0)	0	0.0 (0.0, 0.0)
Education				
No high school diploma or GED	624	9.4 (8.6, 10.2)	416	9.5 (8.4, 10.5)
High school graduate (diploma, GED)	2813	28.2 (27.2, 29.3)	2002	28.2 (26.9, 29.6)
Some college or Associate's degree	3896	27.2 (26.2, 28.1)	2773	27.1 (25.9, 28.3)
Bachelor's degree	3133	19.8 (19.0, 20.6)	2337	20.1 (19.1, 21.1)
Master’s degree or higher	2481	15.4 (14.7, 16.1)	1857	15.1 (14.2, 15.9)
Non-response	0	0.0 (0.0, 0.0)	0	0.0 (0.0, 0.0)
Household Income				
Less than $10,000	371	3.9 (3.4, 4.4)	233	3.9 (3.2, 4.5)
$10,000 to $24,999	1078	9.0 (8.3, 9.6)	727	8.9 (8.1, 9.8)
$25,000 to $49,999	2232	17.0 (16.2, 17.9)	1617	17.0 (15.9, 18.0)
$50,000 to $74,999	2236	16.3 (15.5, 17.2)	1631	16.3 (15.3, 17.4)
$75,000 to $99,999	1999	13.2 (12.5, 13.9)	1499	13.2 (12.3, 14.1)
$100,000 to $149,999	2410	17.9 (17.0, 18.7)	1734	17.9 (16.8, 18.9)
$150,000 or more	2621	22.7 (21.7, 23.6)	1944	22.8 (21.6, 23.9)
Non-response	0	0.0 (0.0, 0.0)	0	0.0 (0.0, 0.0)
Employment				
Working-as a paid employee	6213	53.8 (52.7, 54.9)	4291	52.9 (51.6, 54.3)
Working-self-employed	1048	8.0 (7.4, 8.6)	709	7.2 (6.5, 8.0)
Not working-on temporary layoff from a job	53	0.6 (0.4, 0.8)	35	0.5 (0.3, 0.7)
Not working-looking for work	411	5.2 (4.6, 5.8)	272	5.2 (4.4, 5.9)
Not working-retired	4231	21.0 (20.3, 21.8)	3367	21.3 (20.4, 22.2)
Not working-disabled	417	4.2 (3.7, 4.7)	286	4.5 (3.9, 5.2)
Not working-other	574	7.2 (6.6, 7.9)	425	8.3 (7.4, 9.2)
Non-response	0	0.0 (0.0, 0.0)	0	0.0 (0.0, 0.0)
Census division				
New England	509	4.7 (4.2, 5.2)	374	4.7 (4.1, 5.3)
Mid-Atlantic	1407	12.5 (11.8, 13.3)	1001	12.6 (11.6, 13.5)
East-North Central	1878	14.3 (13.5, 15.0)	1370	14.3 (13.3, 15.2)
West-North Central	952	6.4 (5.9, 6.9)	676	6.4 (5.8, 7.0)
South Atlantic	2538	20.5 (19.6, 21.4)	1881	20.5 (19.4, 21.6)
East-South Central	737	5.8 (5.3, 6.3)	538	5.8 (5.1, 6.5)
West-South Central	1371	11.9 (11.1, 12.7)	965	11.9 (10.9, 12.8)
Mountain	1125	7.7 (7.1, 8.2)	825	7.6 (6.9, 8.3)
Pacific	2430	16.3 (15.5, 17.1)	1755	16.3 (15.3, 17.3)
Non-response	0	0.0 (0.0, 0.0)	0	0.0 (0.0, 0.0)

^*^Values are as of 2022

### Democracy and the Potential for Violence

More than 60% of respondents in 2023 (62.3%, 95% CI 60.9%, 63.7%) perceived “a serious threat to our democracy,” and 84.6% (95% CI 83.4%, 85.7%) considered it very or extremely important “for the United States to remain a democracy”—decreases from 2022 of approximately 5% in both cases (Table 2). About 1 respondent in 6 (16.1%, 95% CI 15.0%, 17.1%) agreed strongly or very strongly that “having a strong leader for America is more important than having a democracy,” a 2.3% decrease from 2022. Strong or very strong agreement with the statement that “the 2020 election was stolen from Donald Trump, and Joe Biden is an illegitimate president” (16.7%, 95% CI 15.7%, 17.7%) did not change significantly from 2022 to 2023.

**Table 2:** Beliefs concerning democracy in the USA

Statement	2022 Respondents (*n* = 12,947)		2023 Respondents (*n* = 9385)		Mean Difference,* 2022–2023	
	Unweighted *n*	Weighted % (95% CI) *Mean* *score* *(95%* *CI)*	Unweighted *n*	Weighted % (95% CI) *Mean* *score* *(95%* *CI)*	Unweighted *n*	Weighted % (95% CI) *Mean* *score* *(95%* *CI)*
*When thinking about democracy in the United States these days, do you believe…*						
There is a serious threat to our democracy. (1)	9409	67.4 (66.3, 68.5)	6452	62.3 (60.9, 63.7)	9385	− 5.2 (− 6.6, − 3.8)
There may be a threat to our democracy, but it is not serious. (2)	2640	23.5 (22.5, 24.5)	2253	28.0 (26.8, 29.3)	9385	4.7 (3.3, 6.2)
There is no threat to our democracy. (3)	780	7.7 (7.0, 8.4)	529	7.0 (6.2, 7.8)	9385	− 0.7 (− 1.8, 0.3)
Non-response	118	1.4 (1.1, 1.7)	151	2.6 (2.1, 3.1)	9385	1.2 (0.7, 1.8)
*Item* *score†*	*12,829*	*1.39* *(1.38,* *1.41)*	*9234*	*1.43* *(1.41,* *1.45)*	*9194*	*0.041* *(0.022,* *0.061)*
*How important do you think it is for the United States to remain a democracy?*						
Not important (1)	191	2.1 (1.8, 2.5)	261	4.0 (3.4, 4.6)	9385	1.8 (1.2, 2.5)
Somewhat important (2)	659	7.7 (7.0, 8.4)	570	9.7 (8.8, 10.7)	9385	2.2 (1.2, 3.3)
Very or extremely important (3)	12,003	89.0 (88.2, 89.8)	8448	84.6 (83.4, 85.7)	9385	− 4.6 (− 5.6, − 3.5)
Non-response	94	1.2 (0.9, 1.4)	106	1.7 (1.3, 2.2)	9385	0.5 (0.0, 0.9)
*Item* *score†*	*12,853*	*2.88* *(2.87,* *2.89)*	*9279*	*2.82* *(2.80,* *2.84)*	*9241*	− *0.064* *(*− *0.078,* − *0.051)*
*Democracy is the best form of government*						
Do not agree (1)	595	5.8 (5.2, 6.4)	531	7.5 (6.7, 8.4)	9385	2.0 (1.1, 2.9)
Somewhat agree (2)	2396	23.1 (22.1, 24.1)	1765	24.1 (22.8, 25.3)	9385	1.1 (− 0.4, 2.5)
Strongly or very strongly agree (3)	9823	69.5 (68.5, 70.6)	6948	65.9 (64.5, 67.3)	9385	− 3.9 (− 5.3, − 2.5)
Non-response	133	1.6 (1.3, 1.9)	141	2.5 (2.0, 3.0)	9385	0.8 (0.3, 1.3)
*Item* *score†*	*12,814*	*2.65* *(2.63,* *2.66)*	*9244*	*2.60* *(2.58,* *2.62)*	*9191*	− *0.057* *(*− *0.075,* − *0.039)*
*These days, American democracy only serves the interest of the wealthy and powerful*						
Do not agree (1)	3976	26.3 (25.4, 27.2)	2789	25.8 (24.6, 26.9)	9385	− 1.0 (− 2.4, 0.3)
Somewhat agree (2)	4499	36.1 (35.0, 37.2)	3678	39.6 (38.2, 40.9)	9385	3.3 (1.6, 5.0)
Strongly or very strongly agree (3)	4354	36.2 (35.1, 37.3)	2781	32.2 (30.9, 33.5)	9385	− 3.2 (− 4.7, − 1.7)
Non-response	118	1.4 (1.1, 1.7)	137	2.4 (1.9, 3.0)	9385	0.9 (0.5, 1.4)
*Item* *score†*	*12,829*	*2.10* *(2.08,* *2.12)*	*9248*	*2.07* *(2.04,* *2.09)*	*9199*	− *0.020* *(*− *0.044,* *0.003)*
*Having a strong leader for America is more important than having a democracy*						
Do not agree (1)	7921	56.2 (55.1, 57.3)	6219	59.6 (58.2, 61.0)	9385	3.0 (1.6, 4.4)
Somewhat agree (2)	2628	23.0 (22.1, 24.0)	1685	21.7 (20.5, 22.9)	9385	− 1.5 (− 3.1, 0.0)
Strongly or very strongly agree (3)	2254	19.1 (18.2, 20.0)	1333	16.1 (15.0, 17.1)	9385	− 2.3 (− 3.6, − 1.1)
Non-response	144	1.6 (1.3, 2.0)	148	2.6 (2.1, 3.2)	9385	0.8 (0.3, 1.3)
*Item* *score†*	*12,803*	*1.62* *(1.60,* *1.64)*	*9237*	*1.55* *(1.53,* *1.57)*	*9182*	− *0.057* *(*− *0.079,* − *0.035)*
*The 2020 election was stolen from Donald Trump, and Joe Biden is an illegitimate president*						
Do not agree (1)	8442	66.9 (65.8, 67.9)	6135	66.7 (65.4, 68.0)	9385	− 1.0 (− 1.9, 0.0)
Somewhat agree (2)	1830	13.5 (12.8, 14.3)	1364	14.1 (13.1, 15.1)	9385	1.0 (0.0, 2.1)
Strongly or very strongly agree (3)	2502	17.9 (17.0, 18.7)	1729	16.7 (15.7, 17.7)	9385	− 0.9 (− 1.8, 0.0)
Non-response	173	1.7 (1.4, 2.0)	157	2.5 (2.0, 3.0)	9385	0.9 (0.4, 1.4)
*Item* *score†*	*12,774*	*1.50* *(1.48,* *1.52)*	*9228*	*1.49* *(1.47,* *1.51)*	*9164*	*0.001* *(− 0.014,* *0.015)*

^*^Among respondents to both surveys (*n* = 9385)

^†^Mean scores in 2022 and 2023 were scored using values indicated in the response lines for individual items. Non-responses were excluded from mean score calculations and differences in mean scores were computed in the subsample of respondents with non-missing responses in both years by computing within-individual change scores and averaging them, to account for the longitudinal study design. For computing differences in individual response levels, indicator variables were computed for each item for each response level and within-individual differences in these were computed and averaged in the subsample of respondents who responded to the survey in both years. This explains why the unweighted n for the mean differences varies

There were decreases from 2022 to 2023 in the proportions of respondents agreeing strongly or very strongly with 2 of 3 statements about conditions in the USA justifying force or violence (Table 3): to “protect American democracy” if “elected leaders will not” [9.7% (95% CI 8.8%, 10.5%) in 2023, a decrease of 8.8%], and to save “our American way of life,” which is “disappearing” [12.1% (95% CI 11.1%, 13.0%) in 2023, a decrease of 3.6%].

**Table 3:** Beliefs concerning the potential need for violence in the USA

Statement	2022 Respondents (*n* = 12,947)		2023 Respondents (*n* = 9385)		Mean Difference,* 2022–2023	
	Unweighted *n*	Weighted % (95% CI) *Mean* *score* *(95%* *CI)*	Unweighted *n*	Weighted % (95% CI) *Mean* *score* *(95%* *CI)*	Unweighted *n*	Weighted % (95% CI) *Mean* *score* *(95%* *CI)*
*If elected leaders will not protect American democracy, the people must do it themselves, even if it requires taking violent actions*						
Do not agree (1)	6461	50.2 (49.1, 51.3)	5920	62.1 (60.7, 63.4)	9385	11.6 (10.1, 13.0)
Somewhat agree (2)	3838	29.6 (28.6, 30.6)	2397	25.9 (24.7, 27.2)	9385	− 3.3 (− 4.9, -1.7)
Strongly or very strongly agree (3)	2502	18.5 (17.6, 19.4)	919	9.7 (8.8, 10.5)	9385	− 8.8 (− 10.0, − 7.7)
Non-response	146	1.6 (1.3, 2.0)	149	2.4 (1.9, 2.8)	9385	0.6 (0.1, 1.1)
*Item* *score†*	*12,801*	*1.68* *(1.66,* *1.69)*	*9236*	*1.46* *(1.44,* *1.48)*	*9170*	− *0.211* *(*− *0.233,* − *0.190)*
*Our American way of life is disappearing so fast that we may have to use force to save it*						
Do not agree (1)	7360	56.0 (54.9, 57.1)	5733	59.6 (58.2, 61.0)	9385	3.3 (1.9, 4.7)
Somewhat agree (2)	3406	26.7 (25.7, 27.7)	2419	26.0 (24.8, 27.3)	9385	− 0.3 (− 1.8, 1.3)
Strongly or very strongly agree (3)	2032	15.8 (15.0, 16.6)	1101	12.1 (11.1, 13.0)	9385	− 3.6 (− 4.7, − 2.4)
Non-response	149	1.5 (1.2, 1.8)	132	2.3 (1.8, 2.8)	9385	0.6 (0.1, 1.1)
*Item* *score†*	*12,798*	*1.59* *(1.58,* *1.61)*	*9253*	*1.51* *(1.49,* *1.53)*	*9182*	− *0.071* *(*− *0.092,* − *0.050)*
*Because things have gotten so far off track, true American patriots may have to resort to violence in order to save our country*						
Do not agree (1)	9486	72.6 (71.6, 73.6)	6905	71.6 (70.3, 72.9)	9385	− 1.4 (− 2.7, − 0.1)
Somewhat agree (2)	2287	17.8 (16.9, 18.6)	1675	18.5 (17.4, 19.6)	9385	1.3 (0.0, 2.6)
Strongly or very strongly agree (3)	992	7.7 (7.1, 8.3)	670	7.6 (6.8, 8.4)	9385	− 0.1 (− 0.9, 0.8)
Non-response	182	2.0 (1.6, 2.3)	135	2.3 (1.8, 2.8)	9385	0.2 (− 0.3, 0.7)
*Item* *score†*	*12,765*	*1.34* *(1.32,* *1.35)*	*9250*	*1.34* *(1.33,* *1.36)*	*9171*	*0.012* *(− 0.006,* *0.030)*
*In the next few years, there will be civil war in the United States*						
Do not agree (1)	6407	47.6 (46.5, 48.8)	6167	63.2 (61.9, 64.6)	9385	15.1 (13.7, 16.5)
Somewhat agree (2)	4746	36.7 (35.6, 37.7)	2576	28.3 (27.1, 29.6)	9385	− 8.0 (− 9.6, − 6.4)
Strongly or very strongly agree (3)	1604	13.7 (12.9, 14.5)	480	5.7 (5.1, 6.4)	9385	− 7.7 (− 8.7, − 6.6)
Non-response	190	2.0 (1.7, 2.4)	162	2.7 (2.2, 3.2)	9385	0.6 (0.0, 1.1)
*Item* *score†*	*12,757*	*1.65* *(1.64,* *1.67)*	*9223*	*1.41* *(1.39,* *1.43)*	*9149*	* − 0.236* *(− 0.255,* − *0.217)*

^*^Among respondents to both surveys (*n* = 9385)

^†^Mean scores in 2022 and 2023 were scored using values indicated in the response lines for individual items. Non-responses were excluded from mean score calculations and differences in mean scores were computed in the subsample of respondents with non-missing responses in both years by computing within-individual change scores and averaging them, to account for the longitudinal study design. For computing differences in individual response levels, indicator variables were computed for each item for each response level and within-individual differences in these were computed and averaged in the subsample of respondents who responded to the survey in both years. This explains why the unweighted n for the mean differences varies

Strong or very strong agreement with the proposition that “in the next few years, there will be civil war in the United States” also declined [5.7% (95% CI 5.1%, 6.4%) in 2023, a 7.7% decrease] (Table 3).

### American Society and Institutions

Four items explored beliefs on race and ethnicity and the great replacement assertion (Table 4), and 3 suggested an increased prevalence of racist beliefs. Strong or very strong agreement with the statement that “white people benefit from advantages in society that Black people do not have” decreased from 2022 to 2023 [35.6% (95% CI 34.2%, 36.9%) in 2023, a 3.9% decrease], as did strong or very strong agreement with the statement that “having more Black Americans, Latinos, and Asian Americans is good for the country” [39.6% (95% CI 38.2%, 40.9%) in 2023, a 5.0% decrease]. Disagreement with the assertion that “discrimination against whites is as big a problem as discrimination against Blacks and other minorities” fell slightly [48.1% (95% CI 46.7%, 49.5%) in 2023, a 2.2% decrease). An item addressing the “great replacement” belief (Table 4) and 3 items addressing the central elements of QAnon mythology and end-time Christianity (Table 5) showed little or no change.

**Table 4:** Beliefs concerning race and ethnicity and American society

Statement	2022 Respondents (*n* = 12,947)		2023 Respondents (*n* = 9385)		Mean Difference,* 2022–2023	
	Unweighted *n*	Weighted % (95% CI) *Mean* *score* *(95%* *CI)*	Unweighted *n*	Weighted % (95% CI) *Mean* *score* *(95%* *CI)*	Unweighted *n*	Weighted % (95% CI) *Mean* *score* *(95%* *CI)*
*White people benefit from advantages in society that Black people do not have*						
Do not agree (1)	4654	31.6 (30.6, 32.6)	3471	31.7 (30.5, 32.9)	9385	0.2 (− 0.9, 1.3)
Somewhat agree (2)	3665	27.8 (26.8, 28.8)	2828	29.9 (28.7, 31.2)	9385	2.5 (1.0, 3.9)
Strongly or very strongly agree (3)	4508	39.3 (38.2, 40.4)	2925	35.6 (34.2, 36.9)	9385	− 3.9 (− 5.1, − 2.8)
Non-response	120	1.3 (1.1, 1.6)	161	2.8 (2.2, 3.3)	9385	1.2 (0.7, 1.7)
*Item* *score†*	*12,827*	*2.08* *(2.06,* *2.10)*	*9224*	*2.04* *(2.02,* *2.06)*	*9181*	* − 0.042* *(− 0.060,* − *0.025)*
*Discrimination against whites is as big a problem as discrimination against Blacks and other minorities*						
Do not agree (1)	6007	49.5 (48.4, 50.6)	4126	48.1 (46.7, 49.5)	9385	− 2.2 (− 3.4, − 0.9)
Somewhat agree (2)	3071	22.6 (21.7, 23.6)	2444	24.7 (23.6, 25.9)	9385	2.1 (0.7, 3.5)
Strongly or very strongly agree (3)	3759	26.6 (25.6, 27.6)	2682	24.9 (23.7, 26.0)	9385	− 0.9 (− 2.1, 0.3)
Non-response	110	1.2 (1.0, 1.5)	133	2.3 (1.8, 2.8)	9385	0.9 (0.5, 1.4)
*Item* *score†*	*12,837*	*1.77* *(1.75,* *1.79)*	*9252*	*1.76* *(1.74,* *1.78)*	*9210*	*0.009* *(− 0.011,* *0.028)*
*Having more Black Americans, Latinos, and Asian Americans is good for the country*						
Do not agree (1)	2774	18.5 (17.6, 19.3)	2323	20.7 (19.7, 21.8)	9385	2.9 (1.7, 4.0)
Somewhat agree (2)	4595	34.3 (33.2, 35.3)	3429	35.7 (34.4, 37.1)	9385	0.7 (− 0.9, 2.2)
Strongly or very strongly agree (3)	5320	45.1 (44.0, 46.2)	3338	39.6 (38.2, 40.9)	9385	− 5.0 (− 6.4, − 3.7)
Non-response	258	2.2 (1.9, 2.6)	295	4.0 (3.4, 4.5)	9385	1.5 (0.9, 2.1)
*Item* *score†*	*12,689*	*2.27* *(2.26,* *2.29)*	*9090*	*2.20* *(2.18,* *2.22)*	*8979*	* − 0.079* *(− 0.099,* − *0.059)*
*In America, native-born white people are being replaced by immigrants*						
Do not agree (1)	7136	57.9 (56.8, 59.0)	5301	58.5 (57.1, 59.8)	9385	0.4 (− 1.1, 1.9)
Somewhat agree (2)	3483	25.0 (24.1, 26.0)	2099	21.7 (20.6, 22.9)	9385	− 3.0 (− 4.5, − 1.5)
Strongly or very strongly agree (3)	2190	15.7 (14.9, 16.5)	1783	16.4 (15.5, 17.4)	9385	0.8 (− 0.3, 2.0)
Non-response	138	1.4 (1.1, 1.7)	202	3.4 (2.8, 3.9)	9385	1.8 (1.2, 2.3)
*Item* *score†*	*12,809*	*1.57* *(1.55,* *1.59)*	*9183*	*1.57* *(1.54,* *1.59)*	*9121*	* − 0.007* *(− 0.029,* *0.016)*

^*^Among respondents to both surveys (*n* = 9385)

^†^Mean scores in 2022 and 2023 were scored using values indicated in the response lines for individual items. Non-responses were excluded from mean score calculations and differences in mean scores were computed in the subsample of respondents with non-missing responses in both years by computing within-individual change scores and averaging them, to account for the longitudinal study design. For computing differences in individual response levels, indicator variables were computed for each item for each response level and within-individual differences in these were computed and averaged in the subsample of respondents who responded to the survey in both years. This explains why the unweighted n for the mean differences varies

**Table 5:** Beliefs concerning QAnon and biblical “end times”

Statement	2022 Respondents (*n* = 12,947)		2023 Respondents (*n* = 9385)		Mean Difference,* 2022–2023	
	Unweighted *n*	Weighted % (95% CI) *Mean* *score* *(95%* *CI)*	Unweighted *n*	Weighted % (95% CI) *Mean* *score* *(95%* *CI)*	Unweighted *n*	Weighted % (95% CI) *Mean* *score* *(95%* *CI)*
*The government, media, and financial worlds in the U.S. are controlled by a group of Satan-worshipping pedophiles who run a global child sex trafficking operation*						
Do not agree (1)	10,276	75.3 (74.2, 76.3)	7333	73.6 (72.3, 74.9)	9385	-2.4 (-3.5, -1.2)
Somewhat agree (2)	1480	13.5 (12.7, 14.4)	1175	14.8 (13.7, 15.9)	9385	1.8 (0.6, 3.1)
Strongly or very strongly agree (3)	953	8.8 (8.1, 9.4)	681	8.7 (7.9, 9.6)	9385	0.2 (-0.7, 1.1)
Non-response	238	2.4 (2.1, 2.8)	196	2.9 (2.4, 3.4)	9385	0.3 (-0.2, 0.9)
*Item* *score†*	*12,709*	*1.32* *(1.30,* *1.33)*	*9189*	*1.33* *(1.31,* *1.35)*	*9088*	*0.025* *(0.008,* *0.042)*
*There is a storm coming soon that will sweep away the elites in power and restore the rightful leaders*						
Do not agree (1)	9064	68.1 (67.1, 69.2)	6735	68.9 (67.6, 70.3)	9385	0.6 (-0.7, 2.0)
Somewhat agree (2)	2474	19.5 (18.6, 20.4)	1774	19.4 (18.3, 20.5)	9385	0.1 (-1.2, 1.4)
Strongly or very strongly agree (3)	1162	9.8 (9.1, 10.5)	672	8.4 (7.6, 9.2)	9385	-1.3 (-2.2, -0.4)
Non-response	247	2.6 (2.2, 3.0)	204	3.3 (2.7, 3.8)	9385	0.6 (0.0, 1.1)
*Item* *score†*	*12,700*	*1.40* *(1.39,* *1.42)*	*9181*	*1.37* *(1.36,* *1.39)*	*9075*	*-0.020* *(-0.039,* *-0.002)*
*The chaos in America today is evidence that we are living in what the Bible calls “the end times.”*						
Do not agree (1)	7412	54.7 (53.6, 55.8)	5536	56.4 (55.0, 57.7)	9385	0.8 (-0.4, 2.0)
Somewhat agree (2)	3137	24.4 (23.4, 25.4)	2245	23.6 (22.4, 24.8)	9385	-0.1 (-1.4, 1.3)
Strongly or very strongly agree (3)	2225	19.0 (18.1, 19.9)	1453	17.5 (16.4, 18.6)	9385	-1.4 (-2.5, -0.3)
Non-response	173	1.9 (1.5, 2.2)	151	2.6 (2.1, 3.1)	9385	0.6 (0.1, 1.1)
*Item* *score†*	*12,774*	*1.64* *(1.62,* *1.65)*	*9234*	*1.60* *(1.58,* *1.62)*	*9159*	*-0.020* *(-0.038,* *-0.002)*

^*^Among respondents to both surveys (*n* = 9385)

^†^Mean scores in 2022 and 2023 were scored using values indicated in the response lines for individual items. Non-responses were excluded from mean score calculations and differences in mean scores were computed in the subsample of respondents with non-missing responses in both years by computing within-individual change scores and averaging them, to account for the longitudinal study design. For computing differences in individual response levels, indicator variables were computed for each item for each response level and within-individual differences in these were computed and averaged in the subsample of respondents who responded to the survey in both years. This explains why the unweighted n for the mean differences varies

### Political Violence

The view that political violence is usually or always justified “in general” remained uncommon in 2023 (2.2%, 95% CI 1.7%, 2.7%) and did not change significantly from 2022 (Table 6). The proportion of respondents who considered violence to be usually or always justified to advance at least 1 political objective fell to 25.3% (95% CI 24.7%, 26.5%), a 6.8% decrease. Among those objectives considered individually (Tables 6 and 7), the proportion of respondents who considered violence to be usually or always justified decreased in 5 cases: “to preserve an American way of life I believe in,” “to oppose the government when it tries to take private land for public purposes,” “to stop voter intimidation,” “to stop police violence,” “to reinforce the police,” and “to keep borders open.” The largest decrease was for violence to reinforce the police [11.0% (95% CI 10.2%, 11.9%) in 2023, a 7.8% decrease]. There were no increases.

**Table 6:** Justification for political violence, in general and for 9 specific objectives

What do you think about the use of force or violence in the following situations?	2022 Respondents (*n* = 12,947)		2023 Respondents (*n* = 9385)		Mean Difference,* 2022–2023	
	Unweighted *n*	Weighted % (95% CI) *Mean* *score* *(95%* *CI)*	Unweighted *n*	Weighted % (95% CI) *Mean* *score* *(95%* *CI)*	Unweighted *n*	Weighted % (95% CI) *Mean* *score* *(95%* *CI)*
*In general…to advance an important political objective that you support*						
Never justified (1)	10,696	79.6 (78.6, 80.5)	7642	78.1 (76.9, 79.3)	9385	− 1.7 (− 3.0, − 0.5)
Sometimes justified (2)	1966	17.1 (16.2, 18.0)	1560	18.9 (17.7, 20.0)	9385	1.9 (0.7, 3.2)
Usually or always justified (3)	246	2.9 (2.5, 3.4)	136	2.2 (1.7, 2.7)	9385	− 0.6 (− 1.3, 0.0)
Non-response	39	0.4 (0.2, 0.5)	47	0.8 (0.5, 1.1)	9385	0.4 (0.2, 0.7)
*Item* *score†*	*12,908*	*1.23* *(1.22,* *1.24)*	*9338*	*1.23* *(1.22,* *1.25)*	*9325*	*0.009* *(− 0.007,* *0.025)*
Violence is usually or always justified to advance at least 1 political objective^‡^	4386	32.5 (31.5, 33.6)	2361	25.3 (24.1, 26.5)	9385	− 6.8 (− 8.1, − 5.4)
*To return Donald Trump to the presidency this year*^§^						
Never justified (1)	11,552	87.1 (86.3, 87.9)	8453	88.5 (87.5, 89.5)	9338	1.1 (0.1, 2.2)
Sometimes justified (2)	625	6.0 (5.4, 6.6)	375	4.9 (4.2, 5.6)	9338	− 1.0 (− 1.9, − 0.1)
Usually or always justified (3)	616	5.3 (4.8, 5.8)	455	5.8 (5.1, 6.5)	9338	0.3 (− 0.6, 1.1)
Non-response	154	1.6 (1.3, 1.9)	55	0.8 (0.5, 1.1)	9338	− 0.4 (− 0.8, − 0.1)
*Item* *score†*	*12,793*	*1.17* *(1.16,* *1.18)*	*9283*	*1.17* *(1.15,* *1.18)*	*9211*	* − 0.007* *(− 0.023,* *0.010)*
*To stop an election from being stolen*^§^						
Never justified (1)	9516	73.6 (72.6, 74.6)	7235	77.2 (76.0, 78.4)	9338	2.8 (1.5, 4.2)
Sometimes justified (2)	2219	16.7 (15.8, 17.5)	1388	14.8 (13.8, 15.8)	9338	− 1.7 (− 3.0, − 0.5)
Usually or always justified (3)	1065	8.3 (7.7, 8.9)	663	7.3 (6.5, 8.0)	9338	− 0.8 (− 1.7, 0.1)
Non-response	147	1.5 (1.2, 1.8)	52	0.8 (0.5, 1.1)	9338	− 0.3 (− 0.7, 0.1)
*Item* *score†*	*12,800*	*1.34* *(1.32,* *1.35)*	*9286*	*1.30* *(1.28,* *1.31)*	*9223*	* − 0.035* *(− 0.054,* − *0.016)*
*To stop people who do not share my beliefs from voting*^§^						
Never justified (1)	12,178	91.6 (90.9, 92.3)	8852	91.7 (90.8, 92.6)	9338	0.0 (− 0.9, 0.9)
Sometimes justified (2)	428	4.7 (4.1, 5.2)	277	4.8 (4.1, 5.6)	9338	0.2 (− 0.6, 1.1)
Usually or always justified (3)	208	2.4 (2.0, 2.8)	159	2.7 (2.2, 3.2)	9338	0.1 (− 0.5, 0.7)
Non-response	133	1.4 (1.1, 1.7)	50	0.8 (0.5, 1.0)	9338	− 0.3 (− 0.7, 0.0)
*Item* *score†*	*12,814*	*1.10* *(1.09,* *1.11)*	*9288*	*1.10* *(1.09,* *1.12)*	*9227*	*0.004* *(− 0.008,* *0.017)*
*To prevent discrimination based on race or ethnicity*^§^						
Never justified (1)	8438	62.3 (61.2, 63.4)	6929	70.4 (69.1, 71.7)	9338	7.7 (6.1, 9.2)
Sometimes justified (2)	3388	27.1 (26.1, 28.1)	1750	20.3 (19.1, 21.4)	9338	− 6.7 (− 8.2, − 5.2)
Usually or always justified (3)	974	9.0 (8.3, 9.7)	607	8.5 (7.6, 9.4)	9338	− 0.6 (− 1.6, 0.5)
Non-response	147	1.5 (1.2, 1.8)	52	0.8 (0.5, 1.1)	9338	− 0.4 (− 0.8, − 0.1)
*Item* *score†*	*12,800*	*1.46* *(1.44,* *1.47)*	*9286*	*1.38* *(1.36,* *1.39)*	*9216*	* − 0.081* *(− 0.103,* − *0.059)*
*To preserve an American way of life based on Western European traditions*^§^						
Never justified (1)	9329	74.2 (73.2, 75.1)	7267	79.2 (78.1, 80.3)	9338	4.8 (3.5, 6.1)
Sometimes justified (2)	2705	18.6 (17.8, 19.5)	1513	14.4 (13.4, 15.3)	9338	− 4.1 (− 5.3, − 2.8)
Usually or always justified (3)	710	5.3 (4.8, 5.8)	483	5.5 (4.8, 6.2)	9338	− 0.1 (− 0.9, 0.8)
Non-response	203	1.9 (1.6, 2.2)	75	1.0 (0.7, 1.3)	9338	− 0.6 (− 1.0, − 0.2)
*Item* *score†*	*12,744*	*1.30* *(1.29,* *1.31)*	*9263*	*1.26* *(1.24,* *1.27)*	*9159*	* − 0.046* *(− 0.064,* − *0.029)*
*To preserve an American way of life I believe in*^§^						
Never justified (1)	6720	55.7 (54.6, 56.8)	6241	69.8 (68.6, 71.1)	9338	13.2 (11.7, 14.6)
Sometimes justified (2)	4449	31.6 (30.5, 32.6)	2221	20.9 (19.8, 22.1)	9338	− 10.7 (− 12.2, − 9.2)
Usually or always justified (3)	1697	11.9 (11.2, 12.6)	804	8.3 (7.5, 9.0)	9338	− 2.8 (− 3.8, − 1.8)
Non-response	81	0.9 (0.6, 1.1)	72	1.0 (0.7, 1.3)	9338	0.4 (0.0, 0.7)
*Item* *score†*	*12,866*	*1.56* *(1.54,* *1.57)*	*9266*	*1.38* *(1.36,* *1.40)*	*9236*	* − 0.164* *(− 0.184,* − *0.143)*
*To oppose Americans who do not share my beliefs*^§^						
Never justified (1)	11,746	88.5 (87.7, 89.3)	8564	88.7 (87.7, 89.7)	9338	− 0.2 (− 1.2, 0.9)
Sometimes justified (2)	871	7.9 (7.3, 8.6)	526	7.5 (6.7, 8.4)	9338	− 0.5 (− 1.5, 0.5)
Usually or always justified (3)	263	2.8 (2.4, 3.2)	184	2.9 (2.4, 3.5)	9338	0.2 (− 0.4, 0.9)
Non-response	67	0.7 (0.5, 1.0)	64	0.9 (0.6, 1.2)	9338	0.4 (0.1, 0.7)
*Item* *score†*	*12,880*	*1.14* *(1.13,* *1.15)*	*9274*	*1.13* *(1.12,* *1.15)*	*9250*	* − 0.001* *(− 0.015,* *0.013)*
*To oppose the government when it does not share my beliefs*^§^						
Never justified (1)	10,607	80.2 (79.2, 81.1)	7890	82.2 (81.0, 83.3)	9338	2.1 (0.9, 3.3)
Sometimes justified (2)	1859	14.9 (14.1, 15.8)	1107	13.0 (12.0, 14.0)	9338	− 1.9 (− 3.1, − 0.7)
Usually or always justified (3)	338	3.4 (2.9, 3.8)	283	3.9 (3.3, 4.5)	9338	0.2 (− 0.5, 0.9)
Non-response	143	1.5 (1.2, 1.8)	58	0.9 (0.6, 1.1)	9338	− 0.4 (− 0.8, 0.0)
*Item* *score†*	*12,804*	*1.22* *(1.21,* *1.23)*	*9280*	*1.21* *(1.20,* *1.23)*	*9219*	* − 0.018* *(− 0.034,* − *0.001)*
*To oppose the government when it tries to take private land for public purposes*^§^						
Never justified (1)	7870	60.7 (59.6, 61.8)	6336	67.6 (66.2, 68.9)	9338	6.3 (4.8, 7.8)
Sometimes justified (2)	3787	28.3 (27.3, 29.3)	2260	23.4 (22.2, 24.5)	9338	− 4.4 (− 6.0, − 2.9)
Usually or always justified (3)	1141	9.5 (8.8, 10.2)	682	8.2 (7.4, 9.0)	9338	− 1.5 (− 2.5, − 0.5)
Non-response	149	1.5 (1.2, 1.8)	60	0.9 (0.6, 1.2)	9338	− 0.3 (− 0.7, 0.1)
*Item* *score†*	*12,798*	*1.48* *(1.47,* *1.50)*	*9278*	*1.40* *(1.38,* *1.42)*	*9204*	* − 0.078* *(− 0.098,* − *0.058)*

^*^ Among respondents to both surveys (*n* = 9385)

^†^ Mean scores in 2022 and 2023 were scored using values indicated in the response lines for individual items. Non-responses were excluded from mean score calculations and differences in mean scores were computed in the subsample of respondents with non-missing responses in both years by computing within-individual change scores and averaging them, to account for the longitudinal study design. For computing differences in individual response levels, indicator variables were computed for each item for each response level and within-individual differences in these were computed and averaged in the subsample of respondents who responded to the survey in both years. This explains why the unweighted n for the mean differences varies

^‡^ Restricted for each respondent to the 13 items presented to that respondent in both years

^§^ 47 participants who did not answer the question "In general…to advance an important political objective that you support" in 2023 were not asked this question

**Table 7:** Justification for political violence for 8 additional specific objectives*

What do you think about the use of force or violence in the following situations?	2022 Respondents (*n* = 12,947)		2023 Respondents (*n* = 9385)		Mean Difference,† 2022–2023	
	Unweighted *n*	Weighted % (95% CI) *Mean* *score* *(95%* *CI)*	Unweighted *n*	Weighted % (95% CI) *Mean* *score* *(95%* *CI)*	Unweighted *n*	Weighted % (95% CI) *Mean* *score* *(95%* *CI)*
*To stop voter fraud*						
Never justified (1)	4772	73.3 (71.9, 74.7)	7180	77.2 (76.0, 78.4)	4697	3.1 (1.2, 4.9)
Sometimes justified (2)	1023	16.3 (15.2, 17.5)	1292	13.4 (12.4, 14.4)	4697	− 2.5 (− 4.4, − 0.6)
Usually or always justified (3)	624	9.4 (8.5, 10.4)	798	8.5 (7.8, 9.4)	4697	− 0.7 (− 2.1, 0.7)
Non-response	43	1.0 (0.7, 1.4)	68	0.9 (0.7, 1.2)	4697	− 0.1(− 0.3, 0.5)
*Item* *score‡*	*6419*	*1.35* *(1.33,* *1.38)*	*9270*	*1.31* *(1.29,* *1.32)*	*4650*	* − 0.038* *(− 0.065,* − *0.011)*
*To stop voter intimidation*						
Never justified (1)	3847	61.2 (59.7, 62.7)	6478	70.4 (69.1, 71.7)	4641	8.4 (6.2, 10.6)
Sometimes justified (2)	1903	27.9 (26.5, 29.3)	2050	20.8 (19.7, 22.0)	4641	− 7.1 (− 9.2, − 4.9)
Usually or always justified (3)	705	10.3 (9.3, 11.3)	742	7.8 (7.1, 8.6)	4641	− 2.1 (− 3.6, − 0.6)
Non-response	30	0.6 (0.4, 1.0)	68	0.9 (0.7, 1.3)	4641	0.8 (0.3, 1.2)
*Item* *score‡*	*6455*	*1.49* *(1.47,* *1.51)*	*9270*	*1.37* *(1.35,* *1.39)*	*4597*	* − 0.113* *(− 0.143,* − *0.082)*
*To stop police violence*						
Never justified (1)	3114	45.5 (43.9, 47.1)	5493	57.7 (56.3, 59.0)	4666	10.9 (8.6, 13.2)
Sometimes justified (2)	2580	41.0 (39.5, 42.6)	2970	31.5 (30.2, 32.8)	4666	− 8.2 (− 10.6, − 5.8)
Usually or always justified (3)	731	12.7 (11.7, 13.9)	807	9.9 (9.1, 10.9)	4666	− 3.0 (− 4.5, − 1.5)
Non-response	37	0.8 (0.5, 1.1)	68	0.9 (0.7, 1.3)	4666	0.3 (0.0, 0.6)
*Item* *score‡*	*6425*	*1.67* *(1.65,* *1.69)*	*9270*	*1.52* *(1.50,* *1.54)*	*4619*	* − 0.141* *(− 0.171,* − *0.111)*
*To reinforce the police*						
Never justified (1)	2377	42.2 (40.6, 43.8)	4851	58.2 (56.9, 59.6)	4672	14.9 (12.7, 17.1)
Sometimes justified (2)	2661	38.7 (37.2, 40.2)	3279	29.8 (28.6, 31.1)	4672	− 7.8 (− 10.1, − 5.5)
Usually or always justified (3)	1404	18.3 (17.2, 19.5)	1139	11.0 (10.2, 11.9)	4672	− 7.8 (− 9.4, − 6.2)
Non-response	43	0.9 (0.6, 1.2)	69	0.9 (0.7, 1.3)	4672	0.7 (0.2, 1.2)
*Item* *score‡*	*6442*	*1.76* *(1.74,* *1.78)*	*9269*	*1.52* *(1.50,* *1.54)*	*4619*	* − 0.231* *(− 0.262,* − *0.200)*
*To stop illegal immigration*						
Never justified (1)	3733	61.0 (59.4, 62.5)	5757	65.7 (64.3, 66.9)	4658	3.7 (1.7, 5.7)
Sometimes justified (2)	1819	26.5 (25.1, 27.9)	2341	22.3 (21.2, 23.5)	4658	− 5.1 (− 7.1, − 3.0)
Usually or always justified (3)	858	11.5 (10.6, 12.6)	1174	11.1 (10.3, 12.0)	4658	1.1 (− 0.5, 2.6)
Non-response	39	1.0 (0.7, 1.5)	66	0.9 (0.7, 1.3)	4658	0.3 (− 0.1, 0.6)
*Item* *score‡*	*6410*	*1.50* *(1.48,* *1.52)*	*9272*	*1.45* *(1.43,* *1.47)*	*4615*	* − 0.028* *(− 0.057,* *0.002)*
*To keep borders open*						
Never justified (1)	4401	66.2 (64.7, 67.7)	7477	78.0 (76.8, 79.2)	4658	11.4 (9.3, 13.5)
Sometimes justified (2)	1535	24.9 (23.5, 26.3)	1295	14.9 (13.9, 15.9)	4658	− 9.1 (− 11.2, − 7.0)
Usually or always justified (3)	518	8.2 (7.3, 9.1)	495	6.1 (5.5, 6.9)	4658	− 2.6 (− 3.9, − 1.3)
Non-response	44	0.7 (0.5, 1.0)	71	0.9 (0.7, 1.3)	4658	0.3 (− 0.1, 0.8)
*Item* *score‡*	*6454*	*1.42* *(1.39,* *1.44)*	*9267*	*1.27* *(1.26,* *1.29)*	*4624*	* − 0.143* *(− 0.171,* − *0.115)*
*To stop a protest*						
Never justified (1)	3682	57.8 (56.2, 59.3)	6599	72.0 (70.7, 73.2)	4656	12.8 (10.7, 15.0)
Sometimes justified (2)	2396	35.3 (33.8, 36.8)	2233	21.4 (20.4, 22.6)	4656	− 12.6 (− 14.8, − 10.4)
Usually or always justified (3)	376	6.0 (5.3, 6.9)	434	5.6 (5.0, 6.4)	4656	− 0.6 (− 1.7, 0.5)
Non-response	41	0.9 (0.6, 1.3)	72	1.0 (0.7, 1.3)	4656	0.4 (0.0, 0.8)
*Item* *score‡*	*6454*	*1.48* *(1.46,* *1.50)*	*9266*	*1.33* *(1.31,* *1.35)*	*4608*	* − 0.137* *(− 0.164,* − *0.110)*
*To support a protest*						
Never justified (1)	5244	78.4 (77.1, 79.7)	7783	80.5 (79.3, 81.6)	4682	2.1 (0.2, 4.1)
Sometimes justified (2)	935	16.4 (15.2, 17.7)	1174	14.1 (13.2, 15.2)	4682	− 2.6 (− 4.4, − 0.8)
Usually or always justified (3)	246	4.5 (3.9, 5.2)	319	4.5 (3.9, 5.2)	4682	0.0 (− 1.2, 1.1)
Non-response	27	0.6 (0.4, 1.0)	62	0.9 (0.6, 1.2)	4682	0.5 (0.1, 1.0)
*Item* *score‡*	*6425*	*1.26* *(1.24,* *1.27)*	*9276*	*1.23* *(1.22,* *1.25)*	*4641*	* − 0.027* *(− 0.053,* − *0.002)*

^*^These objectives were paired in 2022, with respondents randomized 1:1 to see 1 item in each pair

^†^Among respondents to both surveys (*n* = 9385); 47 participants who did not answer the question "In general…to advance an important political objective that you support" in 2023 were not asked these questions

^‡^Mean scores in 2022 and 2023 were scored using values indicated in the response lines for individual items. Non-responses were excluded from mean score calculations and differences in mean scores were computed in the subsample of respondents with non-missing responses in both years by computing within-individual change scores and averaging them, to account for the longitudinal study design. For computing differences in individual response levels, indicator variables were computed for each item for each response level and within-individual differences in these were computed and averaged in the subsample of respondents who responded to the survey in both years. This explains why the unweighted n for the mean differences varies

**Fig. 1:**
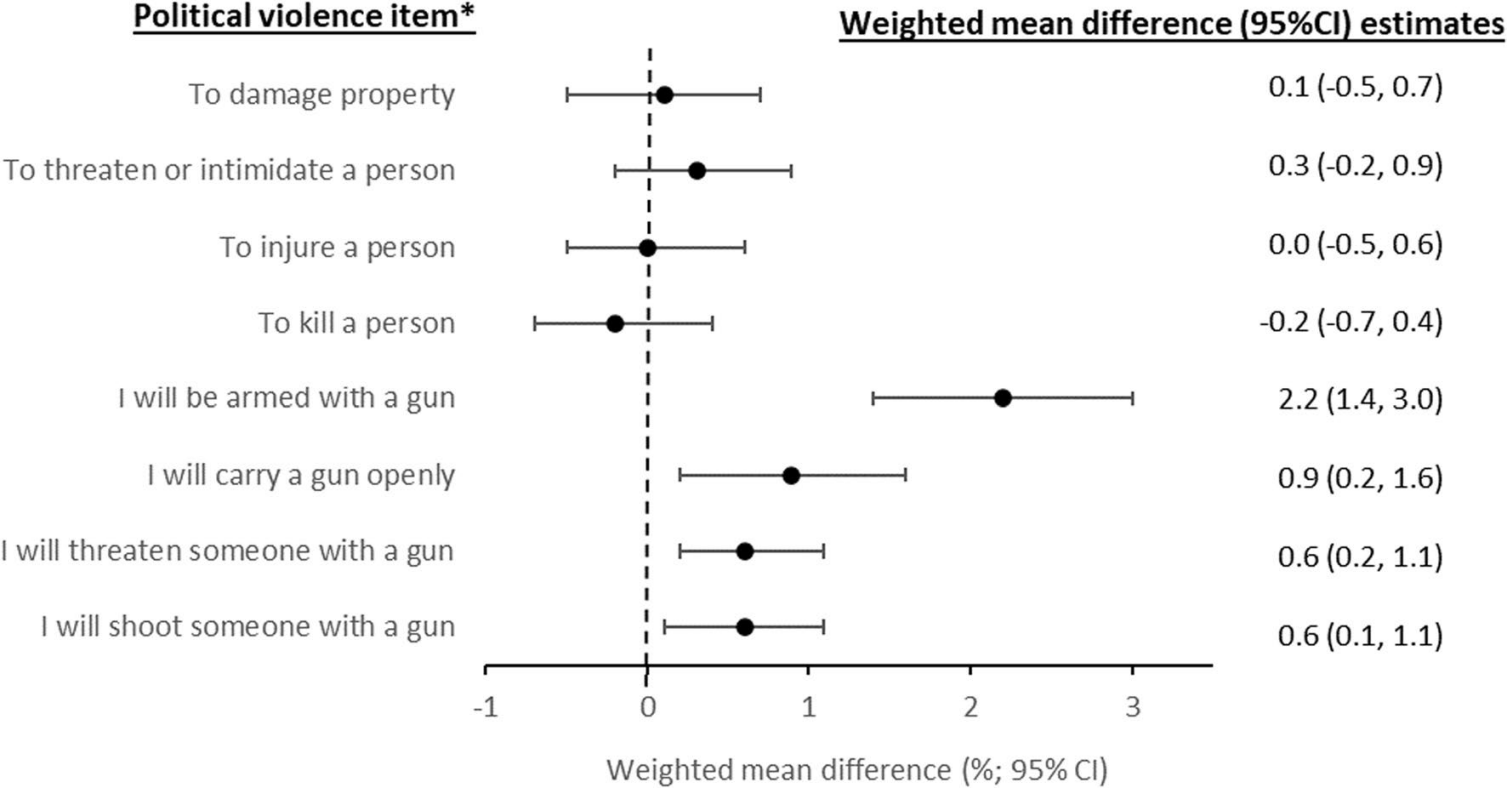
Mean difference in prevalence of willingness to engage in political violence and for firearm involvement. *Items 1–4: Personal willingness to use violence to achieve a political objective (very or completely willing). Items 5–8: Likelihood of using a gun in the future to achieve a political objective (very or extremely likely)

The proportion of respondents who were not asked questions about their personal willingness to use force or violence rose by 7.8% (95% CI 6.3%, 9.2%) (Table 8); this reflects the increase in respondents who did not consider political violence to be at least sometimes justified for any of the 17 specified objectives. Among those asked, the proportions very or completely willing to use force or violence in 2023 remained low and without significant change from 2022: “to damage property,” 3.0% (95% CI 2.5%, 3.6%); “to threaten or intimidate a person,” 2.4% (95% CI 2.0%, 3.0%); “to injure a person,” 2.2% (95% CI 1.8%, 2.7%); “to kill a person,” 1.8% (95% CI 1.5%, 2.3%) (Fig. 1).

**Table 8:** Personal willingness to engage in political violence, by type of violence

In a situation where you think force or violence is justified to advance an important political objective…How willing would *you* *personally* be to use force or violence in each of these ways?	2022 Respondents (*n* = 12,947)		2023 Respondents (*n* = 9385)		Mean Difference,* 2022–2023	
	Unweighted *n*	Weighted % (95% CI) *Mean* *score* *(95%* *CI)*	Unweighted *n*	Weighted % (95% CI) *Mean* *score* *(95%* *CI)*	Unweighted *n*	Weighted % (95% CI) *Mean* *score* *(95%* *CI)*
*To damage property*						
Not asked the question†	2558	21.8 (20.9, 22.8)	2468	30.2 (28.9, 31.5)	9338	7.8 (6.3, 9.2)
Not willing (1)	9101	66.9 (65.8, 68.0)	5856	57.3 (55.9, 58.7)	9338	− 9.2 (− 10.8, − 7.5)
Somewhat willing (2)	920	7.6 (7.0, 8.2)	755	9.0 (8.2, 9.8)	9338	1.3 (0.3, 2.4)
Very or completely willing (3)	303	2.9 (2.5, 3.4)	224	3.0 (2.5, 3.6)	9338	0.1 (− 0.5, 0.7)
Non-response	65	0.7 (0.5, 1.0)	35	0.5 (0.4, 0.8)	9338	− 0.1 (− 0.4, 0.2)
*Item* *score‡*	*10,324*	*1.17* *(1.16,* *1.19)*	*6835*	*1.22* *(1.20,* *1.24)*	*5960*	*0.033* *(0.011,* *0.054)*
*To threaten or intimidate a person*						
Not asked the question†	2558	21.8 (20.9, 22.8)	2468	30.2 (28.9, 31.5)	9338	7.8 (6.3, 9.2)
Not willing (1)	9221	67.8 (66.8, 68.9)	5900	58.5 (57.1, 59.8)	9338	− 8.8 (− 10.5, − 7.2)
Somewhat willing (2)	883	7.5 (6.8, 8.1)	746	8.3 (7.5, 9.1)	9338	0.8 (− 0.1, 1.7)
Very or completely willing (3)	210	2.0 (1.7, 2.4)	177	2.4 (2.0, 3.0)	9338	0.3 (− 0.2, 0.9)
Non-response	75	0.8 (0.6, 1.1)	47	0.6 (0.5, 0.9)	9338	− 0.1 (− 0.4, 0.2)
*Item* *score‡*	*10,314*	*1.15* *(1.14,* *1.16)*	*6823*	*1.19* *(1.17,* *1.21)*	*5942*	*0.025* *(0.005,* *0.046)*
*To injure a person*						
Not asked the question†	2558	21.8 (20.9, 22.8)	2468	30.2 (28.9, 31.5)	9338	7.8 (6.3, 9.2)
Not willing (1)	9374	69.3 (68.3, 70.4)	6137	60.6 (59.2, 62.0)	9338	− 8.4 (− 10.1, − 6.8)
Somewhat willing (2)	709	6.0 (5.4, 6.6)	521	6.2 (5.5, 7.0)	9338	0.6 (− 0.3, 1.4)
Very or completely willing (3)	217	2.0 (1.7, 2.4)	158	2.2 (1.8, 2.7)	9338	0.0 (− 0.5, 0.6)
Non-response	89	0.9 (0.7, 1.1)	54	0.8 (0.6, 1.1)	9338	0.0 (− 0.3, 0.4)
*Item* *score‡*	*10,300*	*1.13* *(1.12,* *1.14)*	*6816*	*1.15* *(1.14,* *1.17)*	*5931*	*0.016* *(− 0.005,* *0.036)*
*To kill a person*						
Not asked the question†	2558	21.8 (20.9, 22.8)	2468	30.2 (28.9, 31.5)	9338	7.8 (6.3, 9.2)
Not willing (1)	9666	71.9 (70.9, 73.0)	6388	63.6 (62.2, 65.0)	9338	− 7.8 (− 9.4, − 6.2)
Somewhat willing (2)	423	3.4 (3.0, 3.9)	292	3.7 (3.1, 4.3)	9338	0.3 (− 0.4, 1.0)
Very or completely willing (3)	225	1.9 (1.6, 2.3)	142	1.8 (1.5, 2.3)	9338	− 0.2 (− 0.7, 0.4)
Non-response	75	0.8 (0.6, 1.1)	48	0.7 (0.5, 1.0)	9338	− 0.1 (− 0.5, 0.3)
*Item* *score‡*	*10,314*	*1.09* *(1.08,* *1.10)*	*6870*	*1.09* *(1.07,* *1.10)*	*5943*	*0.004* *(− 0.015,* *0.022)*

^*^Among respondents to both surveys (*n* = 9385)

^†^These respondents answered “never justified” to all prior questions on the use of force or violence to advance specific political objectives. They were not asked questions on their personal willingness to use political violence

^‡^Mean scores in 2022 and 2023 were scored using values indicated in the response lines for individual items. Non-responses were excluded from mean score calculations and differences in mean scores were computed in the subsample of respondents with non-missing responses in both years by computing within-individual change scores and averaging them, to account for the longitudinal study design. For computing differences in individual response levels, indicator variables were computed for each item for each response level and within-individual differences in these were computed and averaged in the subsample of respondents who responded to the survey in both years. This explains why the unweighted n for the mean differences varies

There were also no significant changes from 2022 to 2023 in the proportions of respondents willing to use force or violence against categories of people defined by their occupations, personal beliefs, or race and ethnicity (Table 9).

**Table 9:** Personal willingness to engage in political violence, by target of violence

In a situation where you think force or violence is justified to advance an important political objective…How willing would *you* *personally* be to use force or violence in each of these ways?	2022 Respondents (*n* = 12,947)		2023 Respondents (*n* = 9385)		Mean Difference,* 2022–2023	
	Unweighted *n*	Weighted % (95% CI) *Mean* *score* *(95%* *CI)*	Unweighted *n*	Weighted % (95% CI) *Mean* *score* *(95%* *CI)*	Unweighted *n*	Weighted % (95% CI) *Mean* *score* *(95%* *CI)*
*An elected federal or state government official*						
Not asked the question†	2558	21.8 (20.9, 22.8)	2468	30.2 (28.9, 31.5)	9338	7.8 (6.3, 9.2)
Not willing	9509	70.5 (69.5, 71.5)	6301	62.0 (60.6, 63.4)	9338	− 8.6 (− 10.2, − 7.0)
Somewhat willing	582	4.6 (4.1, 5.1)	359	4.8 (4.2, 5.6)	9338	0.5 (− 0.4, 1.3)
Very or completely willing	186	1.9 (1.6, 2.3)	129	1.8 (1.4, 2.2)	9338	0.1 (− 0.4, 0.7)
Non-response	112	1.1 (0.9, 1.4)	81	1.2 (0.9, 1.6)	9338	0.2 (− 0.3, 0.6)
*Item* *score‡*	*10,277*	*1.11* *(1.10,* *1.12)*	*6789*	*1.12* *(1.11,* *1.14)*	*5900*	* − 0.014* *(− 0.032,* *0.005)*
*An elected local government official*						
Not asked the question†	2558	21.8 (20.9, 22.8)	2468	30.2 (28.9, 31.5)	9338	7.8 (6.3, 9.2)
Not willing	9582	71.1 (70.1, 72.1)	6347	62.7 (61.4, 64.1)	9338	− 7.8 (− 9.4, − 6.2)
Somewhat willing	515	4.2 (3.7, 4.7)	327	4.2 (3.6, 4.9)	9338	− 0.2 (− 1.0, 0.6)
Very or completely willing	168	1.6 (1.3, 2.0)	118	1.8 (1.4, 2.2)	9338	0.1 (− 0.3, 0.6)
Non-response	124	1.2 (1.0, 1.5)	78	1.1 (0.9, 1.5)	9338	0.1 (− 0.3, 0.5)
*Item* *score‡*	*10,265*	*1.10* *(1.09,* *1.11)*	*6792*	*1.11* *(1.10,* *1.13)*	*5898*	*0.000* *(− 0.017,* *0.017)*
*An election worker, such as a poll worker or vote counter*						
Not asked the question†	2558	21.8 (20.9, 22.8)	2468	30.2 (28.9, 31.5)	9338	7.8 (6.3, 9.2)
Not willing	9874	72.9 (71.9, 73.9)	6507	64.1 (62.7, 65.5)	9338	− 8.3 (− 9.8, − 6.7)
Somewhat willing	283	2.7 (2.3, 3.1)	186	3.0 (2.5, 3.6)	9338	0.3 (− 0.4, 1.0)
Very or completely willing	131	1.5 (1.2, 1.8)	104	1.6 (1.3, 2.1)	9338	0.0 (− 0.5, 0.5)
Non-response	101	1.1 (0.9, 1.4)	73	1.1 (0.8, 1.5)	9338	0.2 (− 0.2, 0.5)
*Item* *score‡*	*10,288*	*1.07* *(1.06,* *1.08)*	*6797*	*1.09* *(1.08,* *1.11)*	*5915*	*0.010* *(− 0.006,* *0.026)*
*A public health official*						
Not asked the question†	2558	21.8 (20.9, 22.8)	2468	30.2 (28.9, 31.5)	9338	7.8 (6.3, 9.2)
Not willing	9750	72.1 (71.0, 73.1)	6433	63.6 (62.2, 64.9)	9338	− 7.8 (− 9.4, − 6.3)
Somewhat willing	386	3.4 (3.0, 3.8)	233	3.3 (2.8, 4.0)	9338	− 0.1 (− 0.9, 0.6)
Very or completely willing	137	1.5 (1.2, 1.9)	126	1.9 (1.5, 2.3)	9338	0.1 (− 0.4, 0.7)
Non-response	116	1.2 (1.0, 1.5)	78	1.1 (0.8, 1.5)	9338	0.1 (− 0.4, 0.5)
*Item* *score‡*	*10,273*	*1.08* *(1.07,* *1.09)*	*6792*	*1.10* *(1.09,* *1.12)*	*5904*	*0.001* *(− 0.015,* *0.018)*
*A member of the military or National Guard*						
Not asked the question†	2558	21.8 (20.9, 22.8)	2468	30.2 (28.9, 31.5)	9338	7.8 (6.3, 9.2)
Not willing	9651	71.2 (70.1, 72.2)	6406	62.9 (61.5, 64.3)	9338	− 7.6 (− 9.2, − 6.0)
Somewhat willing	450	4.0 (3.5, 4.5)	272	4.0 (3.4, 4.6)	9338	− 0.1 (− 0.9, 0.7)
Very or completely willing	180	1.9 (1.6, 2.3)	119	1.9 (1.5, 2.4)	9338	− 0.2 (− 0.7, 0.4)
Non-response	108	1.1 (0.9, 1.4)	73	1.1 (0.8, 1.4)	9338	0.1 (− 0.3, 0.5)
*Item* *score‡*	*10,281*	*1.10* *(1.09,* *1.11)*	*6797*	*1.11* *(1.10,* *1.13)*	*5912*	* − 0.011* *(− 0.030,* *0.008)*
*A police officer*						
Not asked the question†	2558	21.8 (20.9, 22.8)	2468	30.2 (28.9, 31.5)	9338	7.8 (6.3, 9.2)
Not willing	9549	70.3 (69.2, 71.3)	6297	61.3 (59.9, 62.7)	9338	− 8.6 (− 10.2, − 7.0)
Somewhat willing	531	4.6 (4.1, 5.1)	342	5.1 (4.4, 5.9)	9338	0.6 (− 0.2, 1.5)
Very or completely willing	204	2.2 (1.8, 2.6)	152	2.3 (1.9, 2.8)	9338	0.0 (− 0.6, 0.6)
Non-response	105	1.1 (0.9, 1.4)	79	1.1 (0.9, 1.5)	9338	0.2 (− 0.2, 0.6)
*Item* *score‡*	*10,284*	*1.12* *(1.10,* *1.13)*	*6791*	*1.14* *(1.12,* *1.16)*	*5907*	*0.009* *(− 0.010,* *0.028)*
*A person who does not share your race or ethnicity*						
Not asked the question†	2558	21.8 (20.9, 22.8)	2468	30.2 (28.9, 31.5)	9338	7.8 (6.3, 9.2)
Not willing (1)	9865	72.8 (71.8, 73.8)	6477	63.7 (62.3, 65.1)	9338	− 8.5 (− 10.0, − 6.9)
Somewhat willing (2)	290	2.8 (2.4, 3.3)	218	3.4 (2.8, 4.0)	9338	0.5 (− 0.2, 1.3)
Very or completely willing (3)	126	1.5 (1.2, 1.8)	90	1.5 (1.1, 1.9)	9338	− 0.2 (− 0.7, 0.3)
Non-response	108	1.1 (0.8, 1.4)	85	1.3 (1.0, 1.7)	9338	0.3 (− 0.1, 0.7)
*Item* *score‡*	*10,281*	*1.07* *(1.06,* *1.08)*	*6785*	*1.09* *(1.08,* *1.11)*	*5900*	*0.008* *(− 0.008,* *0.023)*
*A person who does not share your religion*						
Not asked the question†	2558	21.8 (20.9, 22.8)	2468	30.2 (28.9, 31.5)	9338	7.8 (6.3, 9.2)
Not willing (1)	9897	73.0 (72.0, 74.0)	6500	63.9 (62.6, 65.3)	9338	− 8.4 (− 9.9, − 6.8)
Somewhat willing (2)	255	2.6 (2.2, 3.1)	194	3.1 (2.5, 3.7)	9338	0.3 (− 0.4, 1.0)
Very or completely willing (3)	117	1.3 (1.0, 1.6)	104	1.7 (1.3, 2.2)	9338	0.4 (− 0.1, 0.9)
Non-response	120	1.3 (1.0, 1.6)	72	1.1 (0.8, 1.4)	9338	− 0.1 (− 0.5, 0.3)
*Item* *score‡*	*10,269*	*1.07* *(1.06,* *1.08)*	*6798*	*1.10* *(1.08,* *1.11)*	*5903*	*0.018* *(0.001,* *0.036)*
*A person who does not share your political beliefs*						
Not asked the question†	2558	21.8 (20.9, 22.8)	2468	30.2 (28.9, 31.5)	9338	7.8 (6.3, 9.2)
Not willing (1)	9757	72.1 (71.1, 73.2)	6417	63.2 (61.9, 64.6)	9338	− 8.3 (− 9.9, − 6.7)
Somewhat willing (2)	403	3.6 (3.1, 4.1)	277	3.9 (3.3, 4.6)	9338	0.3 (− 0.5, 1.0)
Very or completely willing (3)	119	1.3 (1.0, 1.6)	97	1.6 (1.2, 2.1)	9338	0.2 (− 0.3, 0.7)
Non-response	110	1.2 (0.9, 1.4)	79	1.1 (0.8, 1.4)	9338	0.1 (− 0.3, 0.5)
*Item* *score‡*	*10,279*	*1.08* *(1.07,* *1.09)*	*6791*	*1.10* *(1.09,* *1.12)*	*5909*	*0.009* *(− 0.009,* *0.027)*

^*^Among respondents to both surveys (*n* = 9385)

^†^These respondents answered “never justified” to all prior questions on the use of force or violence to advance specific political objectives. They were not asked questions on their personal willingness to use political violence

^‡^Mean scores in 2022 and 2023 were scored using values indicated in the response lines for individual items. Non-responses were excluded from mean score calculations and differences in mean scores were computed in the subsample of respondents with non-missing responses in both years by computing within-individual change scores and averaging them, to account for the longitudinal study design. For computing differences in individual response levels, indicator variables were computed for each item for each response level and within-individual differences in these were computed and averaged in the subsample of respondents who responded to the survey in both years. This explains why the unweighted n for the mean differences varies

However, all predictions that future use of a firearm “in a situation where you think force or violence is justified to advance an important political objective” increased in 2023 (Table 10; Fig. 1); 9.0% (95% CI 8.3%, 9.8%) thought it very or extremely likely that “I will be armed with a gun” (a 2.2% increase), 4.4% (95% CI 3.8%, 5.0%) that “I will carry a gun openly, so that people know I am armed” (a 0.9% increase), 1.6% (95% CI 1.2%, 2.1%) that “I will threaten someone with a gun” (a 0.6% increase), and 1.8% (95% CI 1.4%, 2.2%) that “I will shoot someone with a gun” (a 0.6% increase). Among respondents who considered violence usually or always justified to advance at least 1 specified political objective, about 1 in 20 also thought it very or extremely likely that they would threaten someone with a gun (5.4%, 95% CI 4.0%, 7.0%) or shoot someone (5.7%, 95% CI 4.3%, 7.1%) to advance such an objective (Table S2).

**Table 10:** Future likelihood of firearm possession and use in a situation where political violence is perceived as justified

Thinking now about the future and all the changes it might bring, how likely is it that you will use a gun in any of the following ways in the next few years—in a situation where you think force or violence is justified to advance an important political objective?	2022 Respondents (*n* = 12,947)		2023 Respondents (*n* = 9385)		Mean Difference,* 2022–2023	
	Unweighted *n*	Weighted % (95% CI) *Mean* *score* *(95%* *CI)*	Unweighted *n*	Weighted % (95% CI) *Mean* *score* *(95%* *CI)*	Unweighted *n*	Weighted % (95% CI) *Mean* *score* *(95%* *CI)*
*I will be armed with a gun*						
Not likely (1)	10,408	80.6 (79.7, 81.5)	6832	75.9 (74.7, 77.1)	9385	− 5.6 (− 6.9, − 4.4)
Somewhat likely (2)	1331	10.5 (9.8, 11.3)	1268	12.8 (11.9, 13.8)	9385	2.7 (1.6, 3.8)
Very or extremely likely (3)	1070	7.4 (6.9, 8.0)	1140	9.0 (8.3, 9.8)	9385	2.2 (1.4, 3.0)
Non-response	138	1.4 (1.1, 1.7)	145	2.2 (1.8, 2.7)	9385	0.7 (0.2, 1.2)
*Item* *score†*	*12,809*	*1.26* *(1.24,* *1.27)*	*9240*	*1.32* *(1.30,* *1.33)*	*9181*	*0.076* *(0.059,* *0.094)*
*I will carry a gun openly, so that people know I am armed*						
Not likely (1)	11,559	88.9 (88.2, 89.7)	7992	85.6 (84.6, 86.6)	9385	− 4.1 (− 5.2, − 3.0)
Somewhat likely (2)	751	5.6 (5.1, 6.1)	787	7.6 (6.8, 8.4)	9385	2.4 (1.5, 3.2)
Very or extremely likely (3)	489	3.9 (3.5, 4.4)	451	4.4 (3.8, 5.0)	9385	0.9 (0.2, 1.6)
Non-response	148	1.5 (1.2, 1.8)	155	2.4 (1.9, 2.9)	9385	0.8 (0.3, 1.3)
*Item* *score†*	*12,799*	*1.14* *(1.13,* *1.15)*	*9230*	*1.17* *(1.15,* *1.18)*	*9162*	*0.045* *(0.030,* *0.060)*
*I will threaten someone with a gun*						
Not likely (1)	12,570	96.3 (95.8, 96.7)	8971	93.9 (93.1, 94.7)	9385	− 2.2 (− 3.0, − 1.4)
Somewhat likely (2)	148	1.3 (1.0, 1.6)	168	2.3 (1.8, 2.8)	9385	1.0 (0.4, 1.5)
Very or extremely likely (3)	83	0.9 (0.7, 1.2)	101	1.6 (1.2, 2.1)	9385	0.6 (0.2, 1.1)
Non-response	146	1.5 (1.2, 1.8)	145	2.2 (1.7, 2.7)	9385	0.6 (0.1, 1.1)
*Item* *score†*	*12,801*	*1.03* *(1.03,* *1.04)*	*9240*	*1.06* *(1.05,* *1.07)*	*9172*	*0.024* *(0.014,* *0.034)*
*I will shoot someone with a gun*						
Not likely (1)	12,372	94.8 (94.3, 95.4)	8766	92.3 (91.5, 93.2)	9385	− 2.5 (− 3.4, − 1.6)
Somewhat likely (2)	302	2.6 (2.2, 2.9)	333	3.7 (3.1, 4.3)	9385	1.3 (0.6, 2.0)
Very or extremely likely (3)	132	1.1 (0.9, 1.4)	146	1.8 (1.4, 2.2)	9385	0.6 (0.1, 1.1)
Non-response	141	1.5 (1.2, 1.8)	140	2.2 (1.7, 2.6)	9385	0.6 (0.1, 1.1)
*Item* *score†*	*12,806*	*1.05* *(1.04,* *1.06)*	*9245*	*1.07* *(1.06,* *1.08)*	*9179*	*0.027* *(0.016,* *0.038)*

^*^Among respondents to both surveys (*n* = 9385)

^†^Mean scores in 2022 and 2023 were scored using values indicated in the response lines for individual items. Non-responses were excluded from mean score calculations and differences in mean scores were computed in the subsample of respondents with non-missing responses in both years by computing within-individual change scores and averaging them, to account for the longitudinal study design. For computing differences in individual response levels, indicator variables were computed for each item for each response level and within-individual differences in these were computed and averaged in the subsample of respondents who responded to the survey in both years. This explains why the unweighted n for the mean differences varies

## Discussion

Many of the findings from this second wave of a nationally representative cohort survey in the USA can be viewed as improvements. There were decreases from 2022 to 2023 in perception of a “serious threat” to democracy in that country, in preference for a “strong leader” over a democracy, in expectation that civil war was imminent, in support for general statements of the potential need for violence to address social concerns, and in support for violence to advance specified political objectives. As in 2022, most respondents repeatedly rejected political violence, and most respondents who considered it justified were unwilling to participate in it themselves.

Perhaps criminal convictions and guilty pleas by hundreds of participants in the January 6, 2021 assault on the Capitol (Feuer et al. 2024)—a clear demonstration that such acts can have adverse consequences for the actors—helped decrease support for political violence. Other surveys from 2023 (States United Action 2023; Public Religion Research Institute 2023), which measured political violence differently, have found comparably low levels of support. In 1 case, 51% of respondents considered political violence to be “a major problem.” (States United Action 2023).

This good news comes with a caveat, however. National elections were held in 2022, but not in 2023. Support for political violence may vary with the election cycle, though not all studies have found this to be the case (States United Action 2023). If it does, support for political violence and other measures of polarization will likely increase in 2024.

Other differences in our findings are cause for concern. Support did not decrease for the “Big Lie” that the 2020 election was stolen and for the QAnon delusion. Endorsement of racist beliefs increased slightly. Although support for political violence decreased, willingness to engage in violence among the remaining supporters did not. Personal expectations of firearm use in political violence increased, and in 2023, 5.7% of respondents who believed such violence was usually or always justified to advance at least 1 political objective thought it very likely that they would shoot someone to achieve a political objective.

This good news notwithstanding, public safety, public health, and clinical health professionals will need to collaborate on efforts to prepare for and prevent violent events of a scale that could disrupt critical infrastructure and the 2024 elections and exceed the capabilities of many healthcare delivery systems.

More broadly, there is an urgent need for general public awareness of the threats posed by political violence and for the deployment of preventive interventions beyond those available only to law enforcement agencies. As a recent review of the research literature concluded, it will be important to focus on structural reform and behavior change; intervening on underlying attitudes and beliefs has disappointingly little effect (Kleinfeld 2023). Thoughtful lists of recommendations for policy and social change have been developed (Tisler and Norden 2024; Clapman 2024; Carey et al. 2023; Morales-Doyle et al. 2023). To these should be added, “if you see something, say something”; many prevention measures depend on critical information about threatened violence getting to those in a position to do something about the threat (National Counterterrorism Center 2021). In making that recommendation, we acknowledge that potential reporters may realistically fear that they will face arrest or retaliation, or that those whose conduct they are reporting will be harmed.

## Limitations

Several technical limitations exist. The findings are subject to sampling error and nonresponse bias. Arguably, nonresponse was most important in Wave 1; the 84% response rate for Wave 2 was high*.* A few outcomes are uncommon, with response counts < 100 and weighted prevalences below 5%. The large study sample and small prevalence estimates result in relatively narrow confidence intervals in these cases, but the estimates remain vulnerable to bias from sources such as inattentive or strategic responses. Because 2022 results presented here are for all Wave 1 respondents who responded in Wave 2, and not just those who were included in the Wave 1 main sample, results for 2022 in this report do not replicate those in our initial study (Wintemute et al. 2023).

External events (or their absence) may have affected our findings. In 2022, widely publicized mass shootings occurred in Buffalo, NY and Uvalde, TX while the survey was in the field; there were no comparable events during the fielding of the 2023 survey. The Buffalo shooting is understood to have been a race-related hate crime motivated by great replacement thinking and may have affected respondents’ views on race, violence, and that particular belief. In 2023, the survey closed just before the federal criminal indictment of Donald Trump was handed down; support for violence to return him to the White House increased immediately thereafter (Pape 2023). In both years, Russia’s war against Ukraine may have influenced responses on violence and democracy.

## Conclusions

Findings from this large, nationally representative cohort survey indicate that while support for political violence is common, it is susceptible to change. Planned additional analyses will seek to identify characteristics and life events associated with decreases (and increases) in support for political violence. Increases in expectations of firearm use in political violence are of particular concern. The findings of this analysis will be useful in designing prevention efforts.

### Supplementary Information

**Additional file 1**.

## Data Availability

The datasets generated and/or analyzed during the current study are not publicly available as analyses are continuing but will be made available to qualified researchers subject to the terms of a data use agreement.

## Abbreviations

SD: Standard deviation
CI: Confidence interval
