# Supplementary material for: Single-year change in views of democracy and society and support for political violence in the USA: findings from a 2023 nationally representative survey
Source: Inj Epidemiol. 2024 May 21;11:20. doi: 10.1186/s40621-024-00503-7 (PMC11110245; doi:10.1186/s40621-024-00503-7)
Supplement: Supplementary file 1 — Additional file 1. [file 40621_2024_503_MOESM1_ESM.pdf]

Supplement

Single-Year Change in Views of Democracy and Society and Support for Political Violence in the USA: Findings from a 2023 Nationally Representative Survey

Garen J. Wintemute, MD, MPH*; Sonia L. Robinson, PhD, MPH; Andrew Crawford, PhD; Elizabeth A. Tomsich, PhD; Paul M. Reeping, PhD; Aaron B. Shev, PhD; Bradley Velasquez, MS; Daniel Tancredi, PhD

This supplement has been provided by the authors to give readers additional information about the work.

Page	Title
2	Questions that supplied data for this study
9	References for the question list
10	Additional results text
11	Table S1. Sociodemographic characteristics of respondents and non-respondents in the 2022 and 2023 surveys
13	Table S2. Respondents' views of justification for political violence and personal expectations of firearm use in political violence

QUESTIONS THAT SUPPLIED DATA FOR THIS STUDY

Response options are presented here in order from negative to positive (e.g., “not important” to “extremely important”). Respondents were randomized 1:1 to receive responses in that order or the reverse.

In the list below, questions or items that were repeated or adapted from prior surveys by other investigators contain citations to those surveys.

Domain 1: democracy in the United States

Now we’d like to ask you a few questions about the United States as you see it now, in 2023.

Q: When thinking about democracy in the United States these days, do you believe…?[1]

1. There is a serious threat to our democracy.
2. There may be a threat to our democracy, but it is not serious.
3. There is no threat to our democracy.

Q: How important do you think it is for the United States to remain a democracy?[2]

1. Not important
2. Somewhat important
3. Very important
4. Extremely important

Q: How much do you agree or disagree with the following statements about democracy in the United States?

a. Democracy is the best form of government.[3]

b. These days, American democracy only serves the interests of the wealthy and powerful.[4]

c. Having a strong leader for America is more important than having a democracy.

d. The 2020 election was stolen from Donald Trump, and Joe Biden is an illegitimate president.

1. Do not agree
2. Somewhat agree

3. Strongly agree
4. Very strongly agree

Q: People have different views about violence in the United States. How much do you agree or disagree with each of the following statements?

a. If elected leaders will not protect American democracy, the people must do it themselves, even if it requires taking violent actions.[4]

b. Because things have gotten so far off track, true American patriots may have to resort to violence in order to save our country.[5]

c. Our American way of life is disappearing so fast that we may have to use force to save it.[4]

1. Do not agree
2. Somewhat agree
3. Strongly agree
4. Very strongly agree

Q: How much do you agree or disagree with each of the following statements?

a. In the next few years, there will be civil war in the United States.[6]

1. Do not agree
2. Somewhat agree
3. Strongly agree
4. Very strongly agree

Domain 2: American society and institutions

The next few questions are about your views of American society.

Q: How much do you agree or disagree with each of the following statements about people in the United States today?

a. White people benefit from advantages in society that Black people do not have.[4]

b. Discrimination against whites is as big a problem as discrimination against Blacks and other minorities.[4]

c. A group of people in this country is trying to replace native-born Americans with immigrants and people of color who share their political views. (In 2022: In America, native-born white people are being replaced by immigrants.)

d. Having more Black Americans, Latinos, and Asian Americans is good for the country.[7]

1. Do not agree
2. Somewhat agree
3. Strongly agree
4. Very strongly agree

Q: People have many different views about society in the United States. How much do you agree or disagree with each of the following?

a. The government, media, and financial worlds in the U.S. are controlled by a group of Satan-worshipping pedophiles who run a global child sex trafficking operation.[5]

b. There is a storm coming soon that will sweep away the elites in power and restore the rightful leaders.[5]

c. The chaos in America today is evidence that we are living in what the Bible calls “the end times.”[8]

1. Do not agree
2. Somewhat agree
3. Strongly agree
4. Very strongly agree

Domain 3: violence, including political violence

Now we have a few questions about the use of force or violence. “Force or violence” means physical force strong enough that it could cause pain or injury to a person. A reminder: your responses will be kept confidential and anonymous.

Q: People sometimes talk about using force or violence to achieve political objectives. In general, what do you think about using force or violence to advance an important political objective that you support—is it…?

1. Never justified
2. Sometimes justified
3. Usually justified
4. Always justified

Q: Your view of the use of force or violence to advance an important political objective might depend on the specific objective that was involved. What do you think about the use of force or violence in the following situations—is it never justified, sometimes justified, usually justified, or always justified?

a. To return Donald Trump to the presidency this year

b. To stop an election from being stolen

c. To stop people who do not share my beliefs from voting

d. To prevent discrimination based on race or ethnicity

e. To preserve an American way of life based on Western European traditions

f. To oppose the government when it does not share my beliefs

g. To oppose the government when it tries to take private land for public purposes

1. Never justified
2. Sometimes justified
3. Usually justified
4. Always justified

Q: You said that in general, the use of force or violence was [response inserted] to advance an important political objective that you support. Your opinion might depend on the specific objective that was involved. What do you think about the use of force or violence in the following situations—is it never justified, sometimes justified, usually justified, or always justified?

[Items 1a-4b were paired in 2022, and each respondent was presented with 1 item from each pair. In 2023 all items were presented to all respondents.]

1a. To stop voter fraud

1b. To stop voter intimidation

2a. To stop police violence

2b. To reinforce the police

3a. To stop illegal immigration

3b. To keep our borders open

4a. To stop a protest or demonstration

4b. To support a protest or demonstration

5. To preserve the American way of life l believe in

6. To oppose Americans who do not share my beliefs

1. Never justified
2. Sometimes justified
3. Usually justified
4. Always justified

The next questions are about your personal willingness to use force or violence.

(Questions asked of respondents who endorsed at least 1 use of violence to achieve a specific political objective.)

Q: In a situation where you think force or violence is justified to advance an important political objective, how willing would you personally be to use force or violence in each of these ways?

a. To damage property

b. To threaten or intimidate a person

c. To injure a person

d. To kill a person

1. Not willing
2. Somewhat willing
3. Very willing
4. Completely willing

Q: In a situation where you think force or violence is justified to advance an important political objective, how willing would you personally be to use force or violence against a person because they are…

a. An elected federal or state government official

b. An elected local government official

c. A public health official

d. A member of the military or National Guard

e. A police officer

f. A person who does not share your race or ethnicity

g. A person who does not share your religion

h. An election worker, such as a poll worker or vote counter

i. A person who does not share your political beliefs

1. Not willing
2. Somewhat willing
3. Very willing
4. Completely willing

(Question asked of all respondents.)

Q: Thinking now about the future and all the changes it might bring, how likely is it that you will use a gun in any of the following ways in the next few years—in a situation where you think force or violence is justified to advance an important political objective?

a. I will be armed with a gun.

b. I will carry a gun openly, so that people know I am armed.

c. I will threaten someone with a gun.

d. I will shoot someone with a gun.

1. Not likely
2. Somewhat likely
3. Very likely
4. Extremely likely

REFERENCES FOR THE QUESTION LIST

1. NPR/PBS NewsHour/Marist National Poll. Trust in elections, threat to democracy, November 2021. 2021 November 1. https://maristpoll.marist.edu/polls/npr-pbs-newshour-marist-national-poll-trust-in-elections-threat-to-democracy-biden-approval-november-2021/.

2. Grinnell College National Poll. 52% of Americans believe democracy facing "major threat." Study #2243. 2021 October 20. https://www.grinnell.edu/news/52-americans-believe-democracy-facing-major-threat.

3. The Economist/YouGov Poll. 2021 June 13-16. https://docs.cdn.yougov.com/uagnfc262c/econTabReport.pdf.

4. Survey Center on American Life. January 2021 American Perspectives Survey topline questionnaire. https://www.americansurveycenter.org/wp-content/uploads/2021/03/January-2021-APS-Topline-Questionnaire.pdf.

5. Public Religion Research Institute. The persistence of Q-Anon in the post-Trump era: an analysis of who believes the conspiracies. 2022 Feb 24. https://www.prri.org/research/the-persistence-of-qanon-in-the-post-trump-era-an-analysis-of-who-believes-the-conspiracies/.

6. Zogby. Will the US have another civil war? 2021 Feb 4. https://zogbyanalytics.com/news/997-the-zogby-poll-will-the-us-have-another-civil-war

7. Pew Research Center. Americans see advantages and challenges in country's growing racial and ethnic diversity. 2019 May. https://www.pewresearch.org/social-trends/2019/05/08/americans-see-advantages-and-challenges-in-countrys-growing-racial-and-ethnic-diversity/.

8. IFYC – PRRI Survey on Religion & COVID-19 Vaccine Trust. 2021 March. https://www.prri.org/wp-content/uploads/2021/05/Topline-IFYC-PRRI-Survey-on-Religion-and-COVID-19-Vaccine-Trust-v2_final.pdf.

ADDITIONAL RESULTS TEXT

Three items in the 2023 survey had nonresponse percentages above 3.0%:

Q: People have many different views about society in the United States. How much do you agree or disagree with each of the following?

There is a storm coming soon that will sweep away the elites in power and restore the rightful leaders.

Nonresponse= 3.3%.

Q: How much do you agree or disagree with each of the following statements about people in the United States today?

A group of people in this country is trying to replace native-born Americans with immigrants and people of color who share their political views.

Nonresponse= 3.4%.

Having more Black Americans, Latinos, and Asian Americans is good for the country.

Nonresponse= 4.0%.

Table S1. Sociodemographic characteristics of respondents and non-respondents in the 2022 and 2023 surveys

Characteristic	2022 (Wave1)				Wave1 respondents who left the panel prior to wave2 (n = 1,807)		2023 (Wave2)			
	Respondents (n = 12,947)		Non-respondents (n = 8,318)				Respondents (n = 9,385)		Non-respondents (n = 1,755)	
	Unweighted n	Unweighted %	Unweighted n	Unweighted %	Unweighted n	Unweighted %	Unweighted n	Unweighted %	Unweighted n	Unweighted %
Age										
18-24	488	3.8	1059	12.7	86	4.8	310	3.3	92	5.2
25-34	1309	10.1	1411	17.0	210	11.6	856	9.1	243	13.8
35-44	1884	14.6	1732	20.8	326	18	1252	13.3	306	17.4
45-54	1847	14.3	1599	19.2	335	18.5	1255	13.4	257	14.6
55-64	2794	21.6	1254	15.1	391	21.6	2043	21.8	360	20.5
65-74	2952	22.8	861	10.4	313	17.3	2342	25.0	297	16.9
75+	1673	12.9	402	4.8	146	8.1	1327	14.1	200	11.4
Non-response	0	0.0	0	0.0	0	0.0	0	0.0	0	0.0
Gender										
Male	7158	55.3	3993	48.0	854	47.3	5437	57.9	867	49.4
Female	5789	44.7	4325	52.0	953	52.7	3948	42.1	888	50.6
Non-response	0	0.0	0	0.0	0	0.0	0	0.0	0	0.0
Race and ethnicity										
Black, non-Hispanic	1097	8.5	1039	12.5	170	9.4	749	8.0	178	10.1
Hispanic	1504	11.6	1561	18.8	237	13.1	1016	10.8	251	14.3
White, non-Hispanic	9493	73.3	5030	60.5	1272	70.4	7014	74.7	1207	68.8
Other, non-Hispanic	499	3.9	370	4.4	77	4.3	346	3.7	76	4.3
2+ races, non-Hispanic	354	2.7	318	3.8	51	2.8	260	2.8	43	2.5
Non-response	0	0.0	0	0.0	0	0.0	0	0.0	0	0.0
Marital status										
Now married	8074	62.4	4460	53.6	1089	60.3	5961	63.5	1024	58.3
Widowed	770	5.9	303	3.6	82	4.5	582	6.2	106	6
Divorced	1456	11.2	858	10.3	240	13.3	1010	10.8	206	11.7
Separated	193	1.5	191	2.3	34	1.9	122	1.3	37	2.1
Never married	2454	19.0	2506	30.1	362	20	1710	18.2	382	21.8
Non-response	0	0.0	0	0.0	0	0.0	0	0.0	0	0.0
Education										
No high school diploma or GED	624	4.8	694	8.3	121	6.7	416	4.4	87	5
High school graduate or GED	2813	21.7	2084	25.1	452	25	2002	21.3	359	20.5
Some college or Associate degree	3896	30.1	2649	31.8	584	32.3	2773	29.5	539	30.7
Bachelor's degree	3133	24.2	1726	20.8	372	20.6	2337	24.9	424	24.2
Master's degree or higher	2481	19.2	1165	14.0	278	15.4	1857	19.8	346	19.7
Non-response	0	0.0	0	0.0	0	0.0	0	0.0	0	0.0

Table S1, continued

Characteristic	2022 (Wave1)				Wave1 respondents who left the panel prior to wave2 (n = 1,807)		2023 (Wave2)			
	Respondents (n = 12,947)		Non-respondents (n = 8,318)				Respondents (n = 9,385)		Non-respondents (n = 1,755)	
	Unweighted n	Unweighted %	Unweighted n	Unweighted %	Unweighted n	Unweighted %	Unweighted n	Unweighted %	Unweighted n	Unweighted %
Household Income										
< $10,000	371	2.9	410	4.9	72	4	233	2.5	66	3.8
$10,000 - $24,999	1078	8.3	793	9.5	189	10.5	727	7.7	162	9.2
$25,000 - $49,999	2232	17.2	1558	18.7	318	17.6	1617	17.2	297	16.9
$50,000 - $74,999	2236	17.3	1427	17.2	313	17.3	1631	17.4	292	16.6
$75,000 - $99,999	1999	15.4	1203	14.5	236	13.1	1499	16.0	264	15
$100,000 - $149,999	2410	18.6	1461	17.6	336	18.6	1734	18.5	340	19.4
≥ $150,000	2621	20.2	1466	17.6	343	19	1944	20.7	334	19
Non-response	0	0.0	0	0.0	0	0.0	0	0.0	0	0.0
Employment										
Working full-time	5645	43.6	4514	54.3	889	49.2	3869	41.2	887	50.5
Working part-time	1620	12.5	1342	16.1	258	14.3	1133	12.1	229	13
Not working	5682	43.9	2462	29.6	660	36.5	4383	46.7	639	36.4
Non-response	0	0.0	0	0.0	0	0.0	0	0.0	0	0.0
Census division										
New England	509	3.9	297	3.6	73	4	374	4.0	62	3.5
Mid-Atlantic	1407	10.9	915	11.0	191	10.6	1001	10.7	215	12.3
East-North Central	1878	14.5	1117	13.4	262	14.5	1370	14.6	246	14
West-North Central	952	7.4	597	7.2	137	7.6	676	7.2	139	7.9
South Atlantic	2538	19.6	1652	19.9	326	18	1881	20.0	331	18.9
East-South Central	737	5.7	579	7.0	117	6.5	538	5.7	82	4.7
West-South Central	1371	10.6	1093	13.1	207	11.5	965	10.3	199	11.3
Mountain	1125	8.7	573	6.9	156	8.6	825	8.8	144	8.2
Pacific	2430	18.8	1495	18.0	338	18.7	1755	18.7	337	19.2
Non-response	0	0.0	0	0.0	0	0.0	0	0.0	0	0.0

Mean [(SD) ages were as follows: Wave 1 responders, 55.7 (16.7); Wave 1 non-responders, 45.4 (16.8); Wave 1 respondents who left the panel prior to Wave 2, 52.2 (16.2); Wave 2 responders, 57.0 (16.5); Wave 2 non-responders, 52.5 (17.5).

Table S2. Respondents' views of justification for political violence and personal expectations of firearm use in political violence

**How Likely Is It That You Will Use a Gun in Any of the Following Ways in the Next Few Years—in a Situation Where You Think Force or Violence Is Justified to Advance an Important Political Objective?**	**Violence Is Usually or Always Justified to Advance at Least 1 of 17 Objectives**			
	**No**		**Yes**	
	**Unweighted n**	**Weighted % (95% CI)**	**Unweighted n**	**Weighted % (95% CI)**
I will be armed with a gun.				
Not likely	5262	81.1 (79.9, 82.4)	1570	62.6 (60.1, 66.2)
Somewhat likely	807	10.8 (9.8, 11.8)	461	18.0 (15.9, 20.2)
Very or extremely likely	524	5.6 (5.0, 6.3)	616	17.7 (15.8, 19.5)
Refused	108	2.5 (1.9, 3.1)	37	1.7 (1.0, 2.4)
I will carry a gun openly, so that people know I am armed.				
Not likely	6022	90.3 (89.2, 91.3)	1970	73.6 (71.2, 76.0)
Somewhat likely	429	5.4 (4.6, 6.1)	358	13.2 (11.3, 15.2)
Very or extremely likely	134	1.7 (1.3, 2.2)	317	11.2 (9.5, 12.9)
Refused	116	2.6 (2.0, 3.2)	39	2.0 (1.2, 2.7)
I will threaten someone with a gun.				
Not likely	6519	96.5 (95.8, 97.1)	2452	87.3 (85.2, 89.4)
Somewhat likely	64	1.0 (0.7, 1.3)	104	5.5 (4.0, 7.0)
Very or extremely likely	11	0.2 (0.0, 0.3)	90	5.4 (4.0, 7.0)
Refused	107	2.4 (1.8 2.9)	38	1.8 (1.1, 2.5)
I will shoot someone with a gun.				
Not likely	6433	95.5 (94.7, 96.2)	2333	84.4 (82.2, 86.6)
Somewhat likely	139	1.9 (1.5, 2.3)	194	8.2 (6.5, 9.9)
Very or extremely likely	23	0.2 (0.1, 0.4)	123	5.7 (4.3, 7.1)
Refused	106	2.4 (1.8, 3.0)	34	1.7 (1.0, 2.4)
